# Cross-species single-cell analysis uncovers the immunopathological mechanisms associated with IgA nephropathy progression

**DOI:** 10.1172/jci.insight.173651

**Published:** 2024-05-08

**Authors:** Xizhao Chen, Tiantian Wang, Lei Chen, Yinghua Zhao, Yiyao Deng, Wanjun Shen, Lin Li, Zhong Yin, Chaoran Zhang, Guangyan Cai, Min Zhang, Xiangmei Chen

**Affiliations:** 1 Department of Nephrology, The First Medical Center of Chinese People’s Liberation Army General Hospital, Nephrology Institute of the Chinese People’s Liberation Army, State Key Laboratory of Kidney Diseases, National Clinical Research Center for Kidney Diseases, Beijing Key Laboratory of Kidney Disease Research, Beijing, China.; 2 Department of Critical Care Nephrology and Blood Purification, the First Affiliated Hospital of Xi’an Jiaotong University, Xi’an, China.; 3 Beijing Tsinghua Changgung Hospital, School of Clinical Medicine, Tsinghua University, Beijing, China.; 4 Department of Nephrology, Guizhou Provincial People’s Hospital, Guiyang, China.; 5 Department of Stomatology, The First Medical Center of People’s Liberation Army General Hospital, Beijing, China.

**Keywords:** Autoimmunity, Immunology, Autoimmune diseases, Bioinformatics, Chemokines

## Abstract

IgA nephropathy (IgAN) represents the main cause of renal failure, while the precise pathogenetic mechanisms have not been fully determined. Herein, we conducted a cross-species single-cell survey on human IgAN and mouse and rat IgAN models to explore the pathogenic programs. Cross-species single-cell RNA sequencing (scRNA-Seq) revealed that the IgAN mesangial cells (MCs) expressed high levels of inflammatory signatures CXCL12, CCL2, CSF1, and IL-34 and specifically interacted with IgAN macrophages via the CXCL12/CXCR4, CSF1/IL-34/CSF1 receptor, and integrin subunit alpha X/integrin subunit alpha M/complement C3 (C3) axes. IgAN macrophages expressed high levels of CXCR4, PDGFB, triggering receptor expressed on myeloid cells 2, TNF, and C3, and the trajectory analysis suggested that these cells derived from the differentiation of infiltrating blood monocytes. Additionally, protein profiling of 21 progression and 28 nonprogression IgAN samples revealed that proteins CXCL12, C3, mannose receptor C-type 1, and CD163 were negatively correlated with estimated glomerular filtration rate (eGFR) value and poor prognosis (30% eGFR as composite end point). Last, a functional experiment revealed that specific blockade of the Cxcl12/Cxcr4 pathway substantially attenuated the glomerulus and tubule inflammatory injury, fibrosis, and renal function decline in the mouse IgAN model. This study provides insights into IgAN progression and may aid in the refinement of IgAN diagnosis and the optimization of treatment strategies.

## Abstract 2

We presented the first report to delineate the single cell transcriptomic characteristics of IgA nephropathy (IgAN) across species and identified the key cell players, molecular pathways associated with IgAN progression

## Graphical abstract

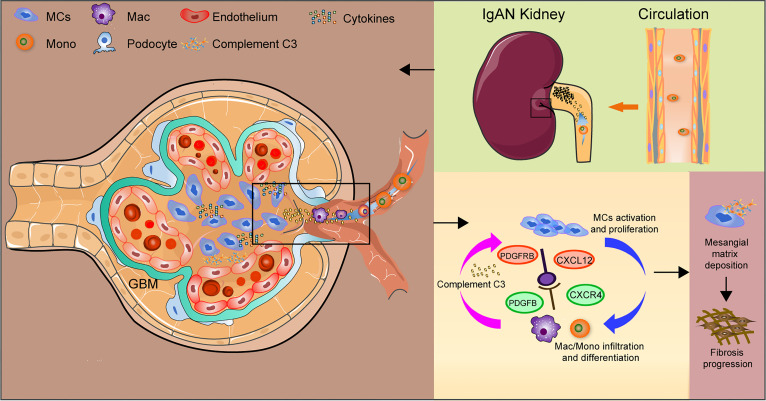

## Introduction

IgA nephropathy (IgAN) is the most common primary glomerulonephritis globally, representing a major cause of kidney failure (1, 2). IgAN is characterized by mesangial hyperplasia and, as such, constitutes a form of mesangial proliferative glomerulonephritis (MsPGN) (3). To date, specific and effective treatments for IgAN have been lacking. Moreover, 30%–40% of patients with IgAN reach end-stage renal disease within 20 years of diagnosis, posing a considerable challenge in terms of clinical management.

IgAN is a typical autoimmune disease, with multiple mechanisms such as immunology, genetics, and environmental or nutritional factors contributing to its pathogenesis (4). The hallmark of IgAN is the constant deposition of IgA-containing immune complexes in the glomerular mesangium, which induce inflammatory responses and immune dysregulation, leading to glomerulonephritis, mesangial cell (MC) proliferation, inflammatory cell recruitment, and myofibroblast formation. These mediators (5, 6) gradually establish pathological crosstalk in the IgAN microenvironment, resulting in glomerular sclerosis, tubular atrophy, and interstitial fibrosis. The precise mechanisms underlying the pathogenesis and progression of IgAN have not been fully determined, although chronic inflammation, oxidative stress, proteinuria, and abnormal activation of complement are known to contribute (7, 8). Accurate diagnostic and prognostic molecular targets for prevention and treatment are urgently needed to improve the prospects of patients with IgAN.

Single-cell RNA sequencing (scRNA-Seq) has emerged as a powerful technology that allows for the unbiased analysis of cellular characteristics within complex and heterogenous biological tissues (9–12). This method has been widely used to decipher transcriptomic landscapes and provide insights into the molecular features involved in chronic kidney disease and acute kidney injury initiation and progression. Cross-species transcriptomic comparison of human IgAN and mouse models at the single-cell level is particularly essential to uncover the key conserved cellular players and molecular features involved in the pathogenesis and progression of this disease. Murine disease models such as ddY IgAN mice (13) have been widely used for biomedical studies and drug target exploration as ddY mice developed spontaneously IgA-dominant deposition in the glomerular mesangium, as well as proteinuria and MC proliferation, then progressed to a definite proliferative glomerulonephritis (14). Systematic comparison of individual cell types in mouse and human glomerular disease has so far been lacking.

Here, to potentially aid in the development of potential molecular targets for IgAN prevention and treatment, we conducted single-cell cross-species transcriptomic analysis on human IgAN, mouse (ddY) IgAN, and rat Thy1.1 nephritis samples. Our primary goal was to determine the conserved pathological cell types and intercellular crosstalk pathways involved in IgAN initiation and progression. We demonstrated that activation of the CXCL12/CXCR4/complement C3 (C3) pathway correlated with mesangium pathological injury and IgAN progression. Moreover, our independent proteomic data demonstrated that CXCL12 protein was highly expressed in IgAN samples that had undergone progression, while higher CXCL12 levels correlated with poor prognosis in IgAN. Interestingly, analysis of the anti-Thy1.1 MsPGN atlas revealed an inflammatory MC phenotype, which expressed Pdgfrb, Cxcl12, Csf1, and Il34, was associated with the MsPGN injury process. Functional experiments showed the significant role of CXCL12 in macrophage recruitment, proliferation, and expression of CXCR4 and C3. To verify our hypothesis, we used a transgenic mouse model that expresses excessive B cell–activating factor (BAFF), which exhibits features of autoimmune disease, including B cell hyperplasia and hypergammaglobulinemia, and develops IgAN with circulating immune complexes and immunoglobulin deposition in the kidney (15, 16). Specific blockade of the CXCL12/CXCR4 pathway significantly alleviated the degree of inflammatory injury, fibrosis, and decline of renal function in the mouse BAFF model.

## Results

### Global analysis of the cellular architecture of human IgAN.

**Figure 1:**
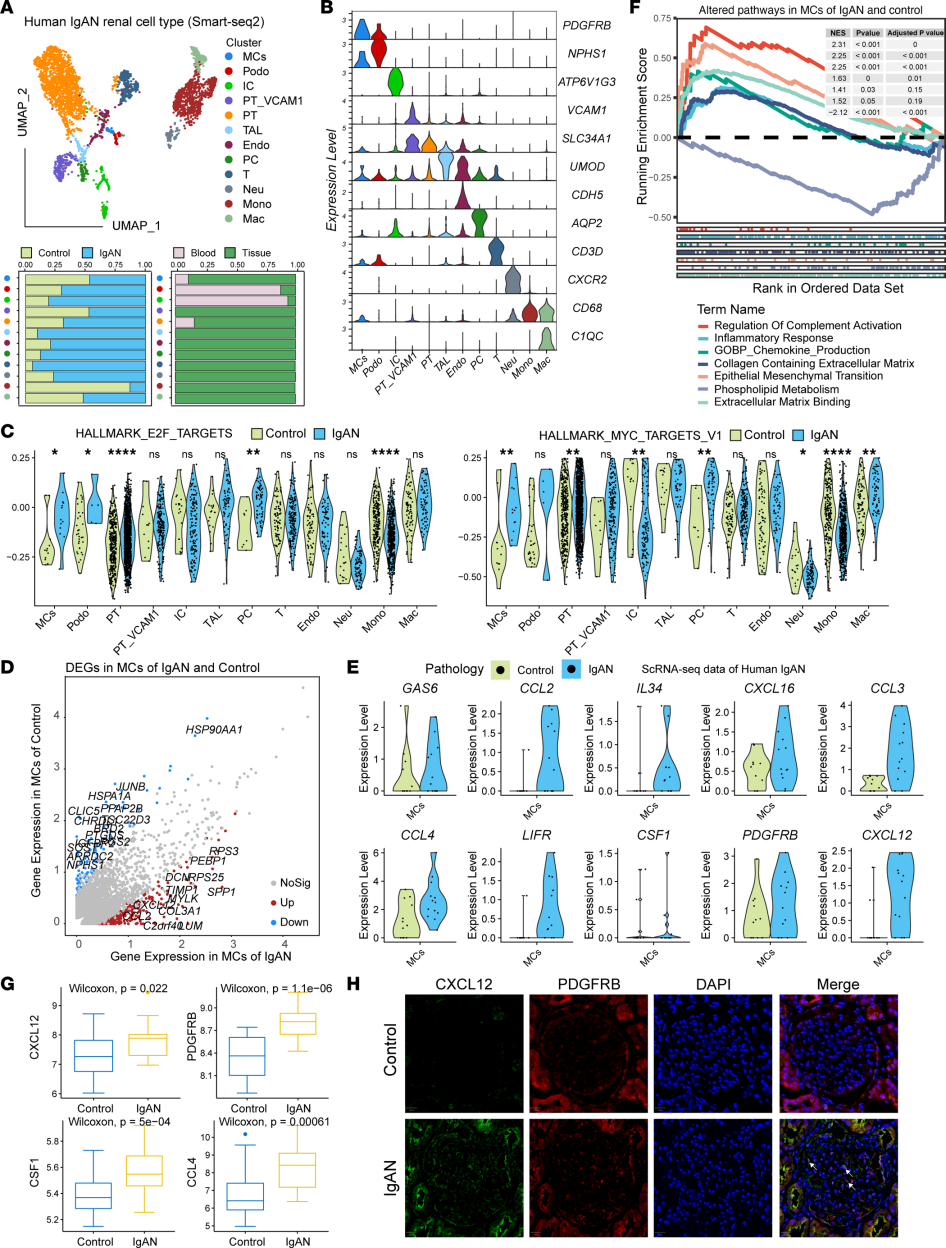
Single-cell transcriptomic architecture in human IgAN. (**A**) Uniform manifold approximation and projection (UMAP) plot showing cell cluster identities and the cell cluster abundance in control and IgAN, as well as in blood and tissue. Endo, endothelial cells; Podo, podocytes; PT, proximal tubular cells; PT_VCAM1, vascular cell adhesion molecule 1–positive PT; TAL, thick ascending limb cells; PC, principal cells; IC, intercalated cells, Neu, neutrophils; T, T cells; Mac, macrophages; Mono, monocytes. (**B**) Violin plots showing the expression of marker genes in each cluster. (**C**) Violin plots showing the expression of proliferation gene sets in each cluster derived from IgAN and normal control. *, *P* < 0.05, **, *P* < 0.01, ****, *P* < 0.0001. Two-tailed *t* test. (**D**) Scatterplots showing gene expression differences in MCs derived from IgAN and control samples. DEGs, differentially expressed genes. (**E**) Violin plots showing gene expression differences in MCs derived from IgAN and control samples. (**F**) GSEA plot showing the enrichment of pathways in MCs derived from IgAN and control samples. NES, normalized enrichment score. (**G**) Box plots showing the expression of CXCL12, C3, CCL4, and CSF1 in bulk IgAN and control RNA-Seq data set; *P* values were determined using the Wilcoxon test. Box plots show the interquartile range, median (line), and minimum and maximum (whiskers). (**H**) Immunostaining showing the coexpression of CXCL12 and PDGFRB in MCs derived from IgAN and control samples. All images in **H** are at original magnification, ×400. The white arrows indicate PDGFRB and CXCL12 coexpressing MCs.

To explore the immunopathogenesis of IgAN, the human tissue and blood samples from IgAN and healthy controls were enrolled to investigate the cellular and molecular characteristics of the human IgAN ecosystem (Supplemental Figure 1; supplemental material available online with this article; https://doi.org/10.1172/jci.insight.173651DS1). We sequenced 3,620 cells and identified and annotated 12 main cell subtypes (Figure 1A and Supplemental Table 1) according to the expression of canonical gene markers (Figure 1B). These subtypes included MCs (marked by *MGP* and *PDGFRB*), endothelial cells (Endo, marked by *PECAM1*, *VWF*, and *CLDN5*), podocytes (Podo, marked by *NPHS2*, *PODXL*, and *PTPRO*), proximal tubular cells (PT, marked by *CUBN*, *SLC13A1*, and *LRP2*), VCAM1-positive PT (PT_VCAM1, marked by *VCAM1*), thick ascending limb cells (TAL, marked by *UMOD* and *SLC12A1*), principal cells (PC, marked by *AQP2* and *AQP3*), intercalated cells (IC, marked by *ATP6V1G3*), neutrophils (Neu, marked by *CXCR2*, *IL1B*), T cells (*CD3D*), macrophages (Mac, marked by *C1QC*), and monocytes (Mono, marked by *CD68*). Cellular composition (Figure 1A) revealed that Neu and Mono were mainly derived from blood samples of patients with IgAN (Figure 1A). As the MCs play an important role in most human glomerulopathies, including IgAN, we mainly explored the phenotype and function difference in MCs of healthy controls and patients with IgAN. We found that proliferation gene set scores were significantly higher in MCs of controls and patients with IgAN (Figure 1C, *P* < 0.05). Interestingly, we found that IgAN-associated MCs expressed high levels of inflammatory and immunoregulatory genes, such as *CXCL12*, *IL34*, and *CSF1*, as well as the chemokine genes *CCL2*, -*3*, and -*4* (Figure 1, D and E). Gene set enrichment analysis (GSEA) of the top 50 upregulated DEGs between IgAN-associated MCs and normal MCs revealed enrichment of PDGFRB, complement, and fibrosis pathways (Figure 1F). Bulk RNA-Seq profiling validated the higher levels of *CXCL12*, *PDGFRB*, *CSF1*, and *CCL4* expression in IgAN samples, compared with healthy controls (Figure 1G). We also performed a multiplex immunostaining experiment, which revealed that CXCL12 was coexpressed with PDGFRB in MCs; moreover, the levels of CXCL12 and PDGFRB proteins were higher in IgAN tissue than in healthy control tissue (Figure 1H). These results delineated the cellular characteristics of IgAN at single-cell resolution and supported the identity of IgAN as a mesangial proliferative autoimmunity glomerulonephritis (MsPGN) arising from abnormal immune activation.

Next, we performed validation analysis using a bulk RNA-Seq IgAN data set consisting of 20 IgAN samples and 22 healthy control samples (17). Deconvolution analysis revealed that the IgAN samples were enriched for MC, Endo, and Mono/Mac types, while the percentages of tubular and collection tube epithelial cell types such as PT, DT, IC, and PC were significantly reduced in IgAN samples, compared with healthy controls (Supplemental Figure 2). DEG analysis revealed that IgAN samples exhibited high expression levels of the matrix and collagen genes *LUM* and *COL1A2* and the immune activation-associated genes *CXCL2,* -*6*, and -*11* and *CX3CR1*. GSEA of the top 50 upregulated IgAN DEGs further supported the abovementioned concept that IgAN is characterized by abnormal activation of immune and fibrosis pathways (Supplemental Figure 2). Overall, these findings indicate the important role of immune modulation in IgAN.

### Contribution of blood monocytes to kidney macrophage infiltration and C3 deposition.

**Figure 2:**
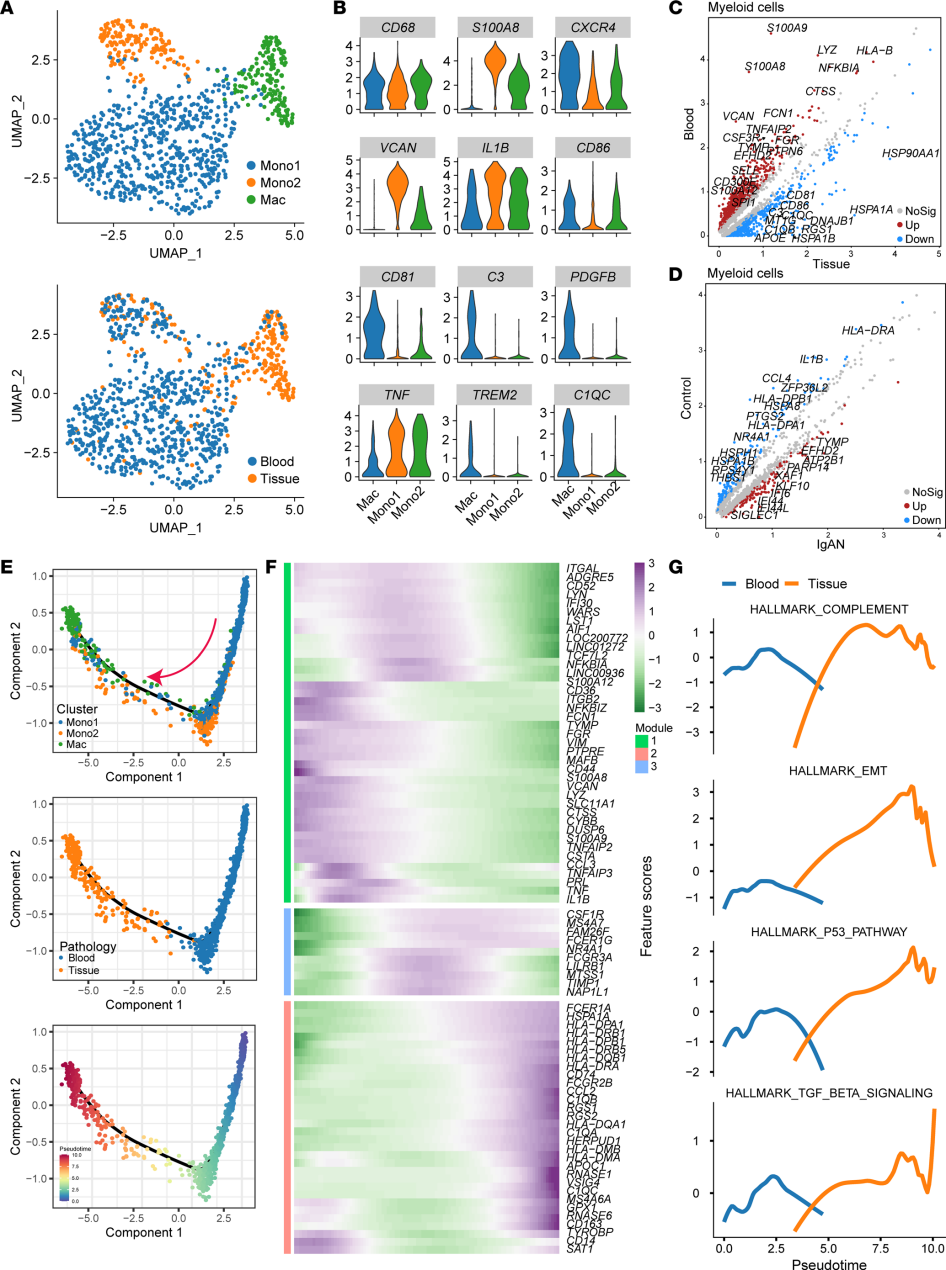
Macrophage/monocyte characteristics and differentiation trajectories in IgAN. (**A**) UMAP plot showing Mono/Mac clusters in IgAN kidney tissue and blood (upper panel) and distribution in pathological samples (lower panel). (**B**) Violin plots showing the expression of M1 macrophage– and IgAN progression–associated macrophage/monocyte marker genes. (**C**) Scatterplots showing gene expression differences in macrophages/monocytes derived from tissue and blood. (**D**) Scatterplots showing gene expression differences in macrophages/monocytes derived from IgAN and control samples. (**E**) Differentiation trajectories of macrophages/monocytes. The pink arrow indicates the cell differentiation trajectory. (**F**) Monocle pseudotemporal gene expression dynamics alongside macrophage/monocyte differentiation trajectories. (**G**) Monocle pseudotemporal pathway dynamics alongside macrophage/monocyte differentiation trajectories.

C3, a key molecule in the complement system, is involved in immune and inflammatory responses and is closely associated with the pathogenesis and progression of IgAN. However, the source of complement C3 and mechanism of C3 protein infiltration/deposition in the glomerular mesangium remain poorly understood. To address these issues, we analyzed IgAN-associated Mono/Mac subpopulations in human IgAN tissue and blood scRNA-Seq data. Subclustering of Mono/Macs generated 1 Mac and 2 Mono clusters (Figure 2A). The macrophages expressed high levels of M1 macrophage markers *CD68*, *IL1B*, and *CD86* in addition to high levels of *PDGFB* and *TNF* (Figure 2, B and C), which have been reported to be important factors for MC activation. In addition, tissue macrophages expressed high levels of *C3*, *C1QC*, and interferon-encoding genes, such as *IFI6/44/44L* (Figure 2D), implying their involvement in IgAN development. We also found that the macrophages were exclusively derived from kidney tissues, while most monocytes were derived from blood of patients with IgAN. We hypothesized that kidney macrophages in IgAN may derive from the infiltration and differentiation of circulating blood monocytes. To verify this possibility, we performed pseudotime-based differentiation trajectory analysis, which indicated that circulating blood monocytes underwent a differentiation route to macrophages from blood to tissue (Figure 2E). The dynamic pseudotemporal expression pattern of specific representative genes also supported the transition of blood monocytes to tissue macrophages (Figure 2F). Pathway analyses revealed upregulation of complement, P53, epithelial-mesenchymal transition (EMT), and TGF-β pathways alongside the monocyte differentiation trajectory from blood to kidney tissue in IgAN (Figure 2G). These findings revealed the potential origin of infiltrating macrophages and depicted the dynamic expression levels of genes and pathways alongside the monocyte differentiation trajectories.

### Cross-species analysis of intercellular interactions in IgAN ecosystem.

**Figure 3:**
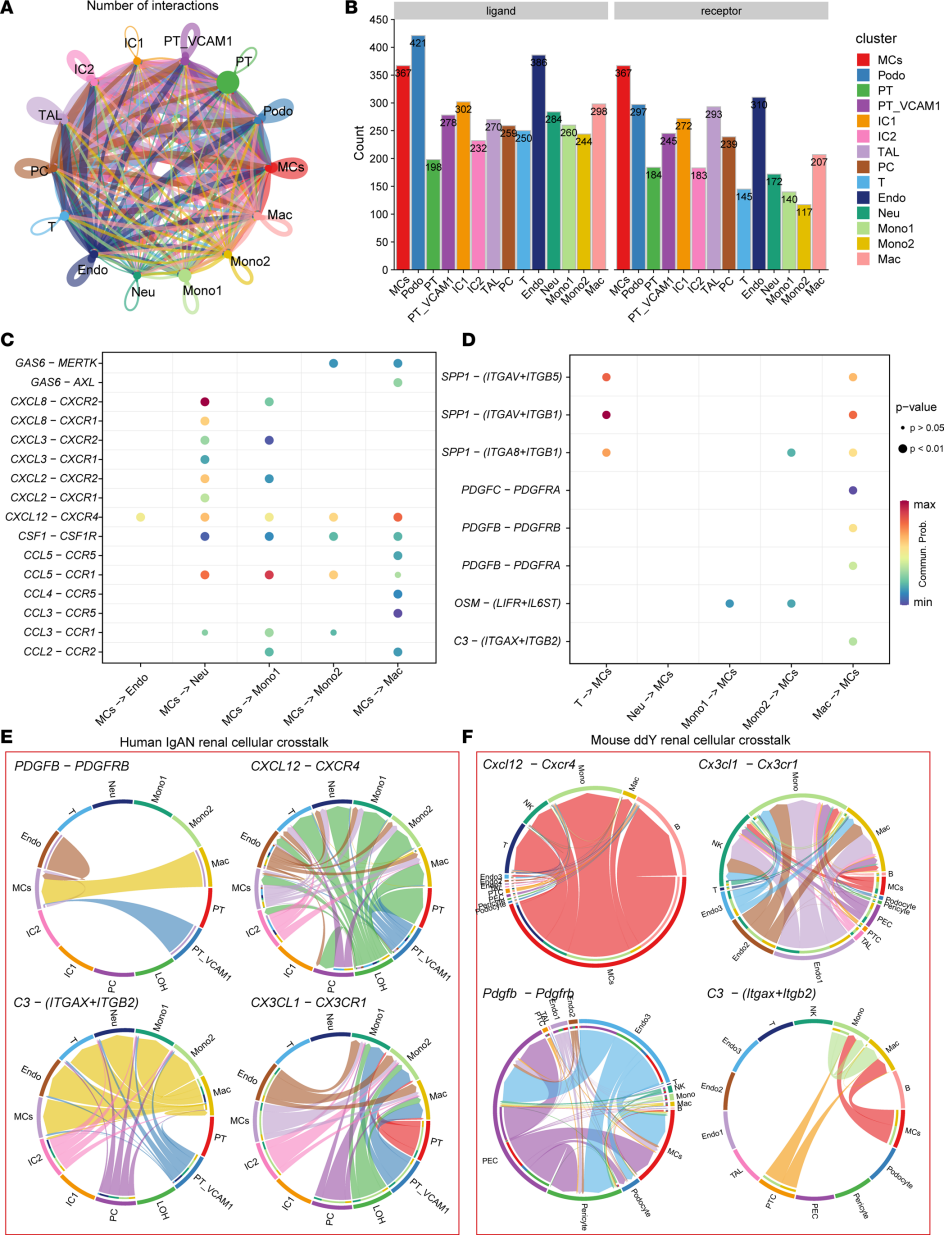
Cross-species single-cell analysis of intercellular crosstalk in human and mouse IgAN. (**A**) Interaction weights/strength among cell types in human IgAN. (**B**) Bar plot showing the significant ligand-receptor pair counts between cell types in human IgAN. (**C** and **D**) Bubble plot showing significant ligand-receptor pairs for immune cells and MCs in human IgAN. Two-tailed *t* test. Commun. Prob., communication probability. (**E** and **F**) Chord diagram showing conserved ligand-receptor pairs for immune cells and MCs in human (**E**) and ddY mouse (**F**) IgAN.

To explore the mechanisms underlying the infiltration of Mono/Macs into kidney tissue and the conserved pathological cellular interactions in IgAN, we conducted intercellular interaction analyses based on ligand-receptor pairs (Figure 3, A and B, and Supplemental Figure 3). Interestingly, interaction weights/strength were higher in MCs than all other cell types (Figure 3, A and B), and MCs expressed more ligands corresponding to receptors expressed by Mono/Macs and MCs than by other cell subtypes (Figure 3B). Intercellular crosstalk analysis revealed that immune and inflammatory signaling pathways such as CXCL, CCL, TNF, GAS, PDGFB, and complement were enriched in MCs and immune cells. Further ligand-receptor analysis revealed that immune signaling axes (Figure 3, C and D, and Supplemental Figure 3) such as *GAS6/AXL*, *CCL2/3/4/5/CCR1/2/5*, *CXCL12/CXCR4*, *PDGFRB/PDGFB*, and *C3/*integrin subunit alpha X*/*integrin subunit beta 2 (*C3*/*ITGAX/ITGB2*) might participate in intercellular crosstalk between MCs and Mono/Macs in human IgAN. Moreover, crosstalk analysis in human IgAN and the ddY IgAN mouse model (3,076 cells) revealed that MCs expressed the ligands *CXCL12*, *PDGFRB*, and *ITGAX/ITGB2*, corresponding to the receptors *CXCR4*, *PDGFB*, and *C3* expressed by macrophages (Figure 3, E and F; Supplemental Figure 4; and Supplemental Table 2). The above results indicated that in IgAN, MCs express and secrete *CXCL12* or *CX3CL1* and may thus recruit circulating blood Mono/Macs into kidney tissue via interactions with *CXCR4* or *CX3CR1* receptors expressed on macrophages. In turn, kidney macrophages can express and secrete *PDGFB* or *C3*, leading to the activation of MCs through *PDGFRB/PDGFB* and *C3/ITGAX/ITGB2* ligand-receptor pairs.

### Proteomic profiling shows significance of CXCL12 in human IgAN progression and prognosis.

**Figure 4:**
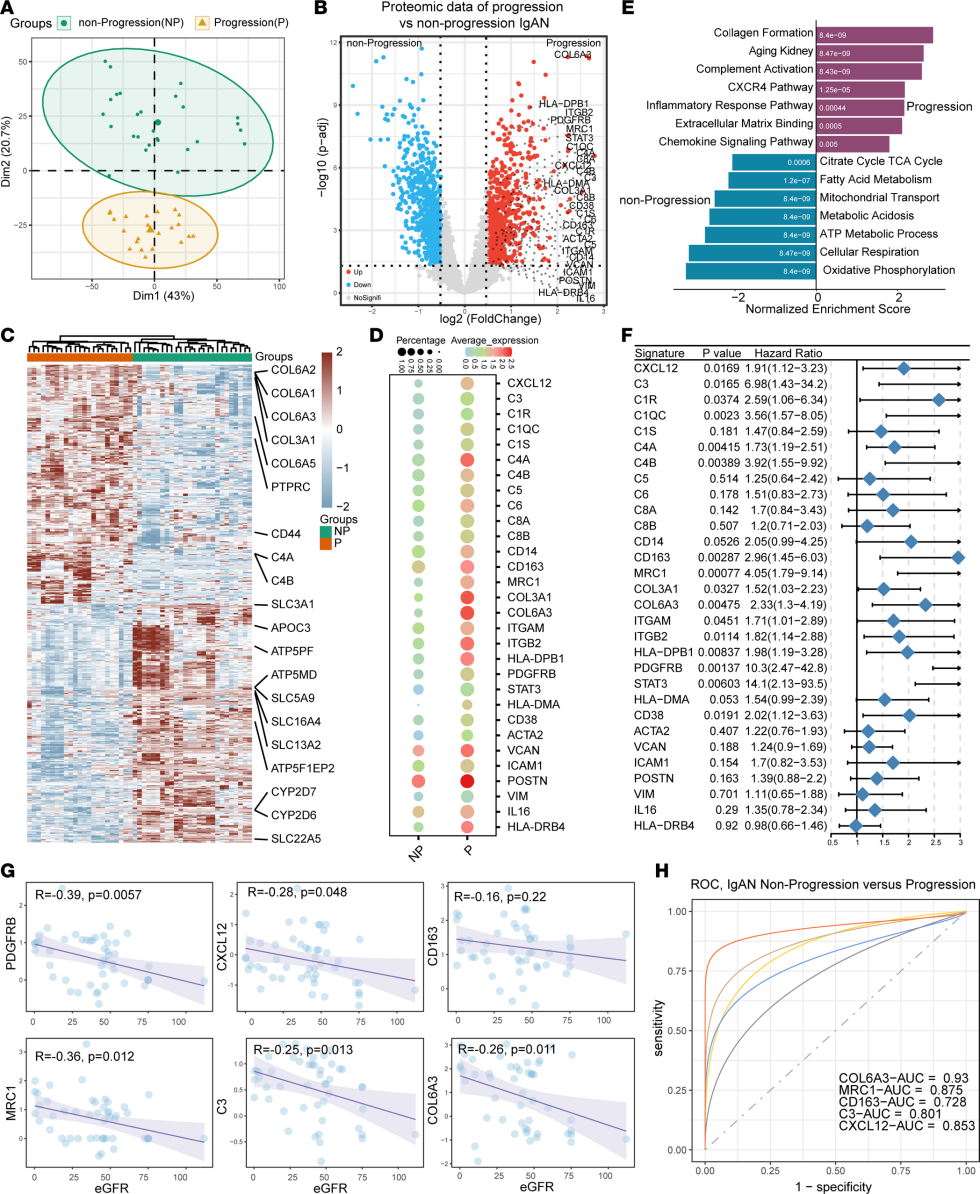
Proteomic profiling showing the significance of CXCL12 in human IgAN progression. (**A**) PCoA plot showing the classification of nonprogression and progression human IgAN samples. (**B**) Volcano map showing the differentially expressed proteins (DEPs) among nonprogression and progression human IgAN samples. (**C**) Heatmap showing the DEPs among nonprogression and progression human IgAN samples. (**D**) Dot plot showing the expression of selected DEPs among nonprogression and progression human IgAN samples. (**E**) GSEA plot showing the enrichment of pathways among nonprogression and progression human IgAN samples. (**F**) Forest plot showing the hazard ratios of the upregulated DEPs in progression human IgAN samples. (**G**) Scatterplot showing the correlation between proteins CXCL12, C3, PDGFRB, COL6A3, MRC1, and CD163 with the eGFR value. (**H**) The diagnostic power of proteins CXCL12, C3, COL6A3, MRC1, and CD163 in classification of nonprogression and progression human IgAN samples. ROC, receiver operating characteristic.

Having defined the pathological cellular model and the intercellular interactions within the IgAN microenvironment, we further validated the key molecular targets identified in scRNA-Seq using bulk IgAN protein-profiling data of 21 IgAN samples showing progression and 28 samples without progression (18), and the detailed clinical data of 49 patients with IgAN are seen in Supplemental Table 3. Principal coordinates analysis (PCoA) was used to explore the differentially expressed proteins (DEPs) of progression and nonprogression IgAN samples (Figure 4A). After strict filtering, 725 DEPs (fold-change [FC] > 0.5, *P* < 0.05) were upregulated in progression versus nonprogression IgAN samples (Figure 4, B and C). Among them, the protein PDGFRB, and collagen proteins such as COL6A3 and COL3A1, as well as a panel of immune and inflammatory proteins such as CXCL12, C3, C4A, C4B, C5, CD163, mannose receptor C-type 1 (MRC1), and CD38 were highly expressed in progression compared with nonprogression IgAN samples (Figure 4D). Further GSEA of the upregulated DEPs revealed that progression IgAN was characterized by inflammatory, complement, and fibrosis signatures (Figure 4E), which indicated that immune and complement activation participate in the progression of IgAN. Furthermore, the proteins PDGFRB, CXCL12, C3, MRC1, COL6A3, and CD163 were correlated with poor IgAN prognosis (30% estimated glomerular filtration rate [eGFR] decline as endpoint, Figure 4F) in the follow-up period and negatively correlated with the eGFR value (Figure 4G), and these proteins achieved a high diagnostic power for classification of progression and nonprogression IgAN (Figure 4H). Analysis of another public chronic kidney disease (CKD) data set (Supplemental Figure 5) also revealed that CXCL12/CXCR4/C3 was highly correlated with the severity of CKD. These results indicated that CXCL12 has an important role in IgAN progression.

### Role of CXCL12 in the proliferation and migration of macrophages.

The above intercellular crosstalk analysis demonstrated that in IgAN, MCs expressed high levels of the ligand CXCL12, while macrophages expressed the corresponding receptor, CXCR4. These findings provided a potential mechanism for the recruitment of macrophages into kidney tissue by MCs, thereby facilitating inflammation and immune injury. To validate this hypothesis, we performed in vitro wound-healing assays using macrophages. Compared with administration of vehicle, stimulation with Cxcl12 resulted in a significantly increased degree of gap closure after 24 hours and 48 hours, indicating enhanced cell migration (Supplemental Figure 6, A and B). We then knocked down (via Cxcl2 siRNA, si-Cxcl12) or overexpressed (via transfection of an overexpression plasmid, OE-Cxcl12) *Cxcl12* in MCs cocultured with macrophages in a Transwell system (Supplemental Figure 7). These coculture experiments revealed that OE-Cxcl12-MCs exhibited an enhanced ability to recruit macrophages, whereas si-Cxcl12-MCs had little effect (Supplemental Figure 6, D and E). Furthermore, 5-ethynyl-2′-deoxyuridine (EdU) assays showed that OE-Cxcl12-MCs significantly enhanced the proliferation of macrophages while si-Cxcl12-MCs had little effect (Supplemental Figure 6F). Meanwhile, OE-Cxcl12-MCs induced significantly elevated expression of Cxcr4 and C3 in macrophages. By contrast, C3 expression decreased significantly in macrophages that were cocultured with si-Cxcl12-MCs (Supplemental Figure 6, G and H). The above results showed that CXCL12 exerted a significant influence on macrophage migration and proliferation, as well as the production of C3 in macrophages. These findings support the role of the CXCL12/CXCR4/C3 pathway in the initiation and progression of IgAN.

### Comprehensive scRNA-Seq survey of anti-Thy1 MsPGN model.

**Figure 5:**
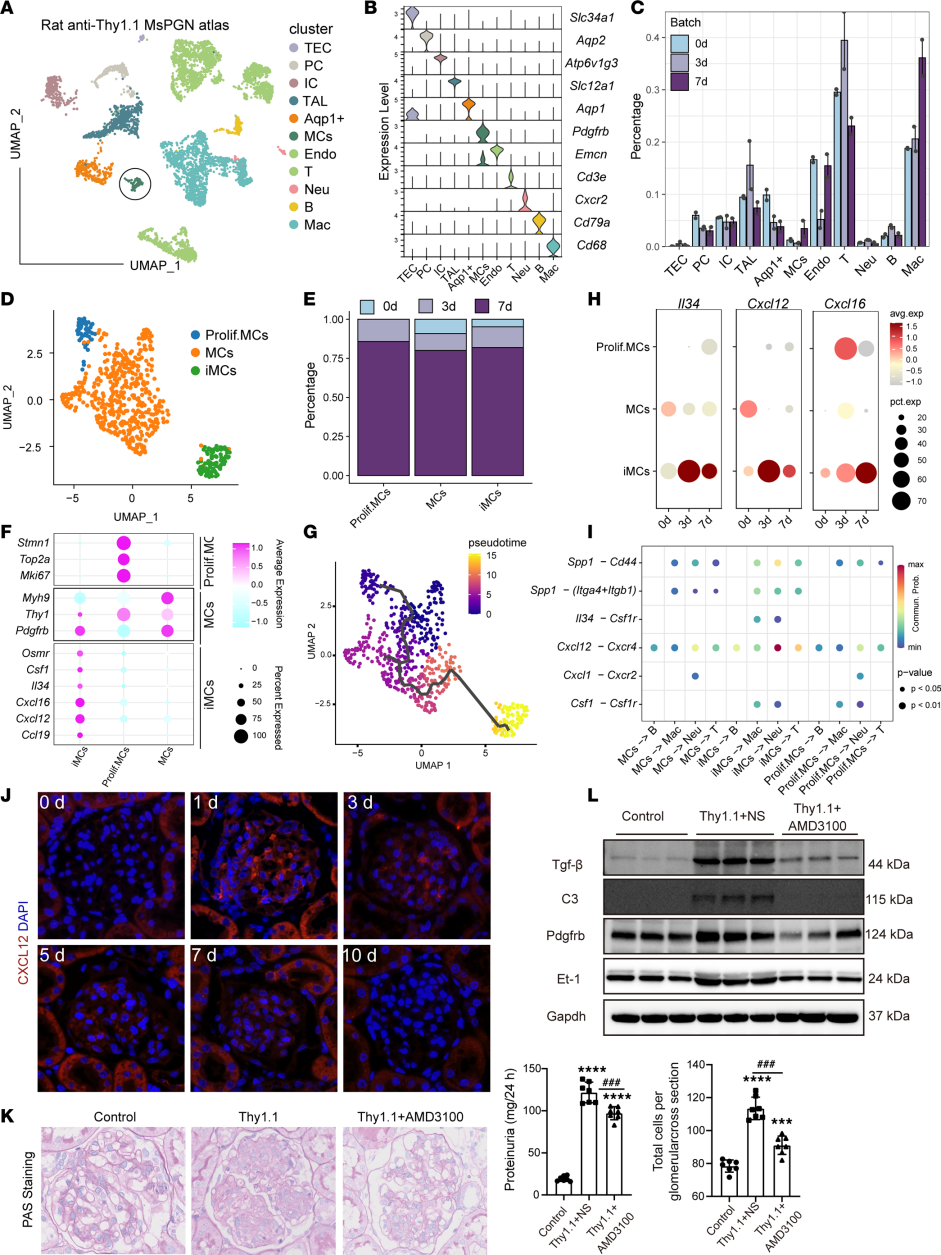
Altered Cxcl12 expression in the rat anti-Thy1.1 MsPGN model. (**A**) UMAP plot showing the cellular landscape of rat anti-Thy1.1 MsPGN model. TEC, tubular epithelial cells; Aqp1, aquaporin 1. (**B**) Violin plots showing the expression of marker genes in each cluster of the rat anti-Thy1.1 MsPGN model. (**C**) The cell cluster percentage distribution at different time points in the rat anti-Thy1.1 MsPGN model. (**D**) UMAP plot showing the MC phenotype diversity and the MC subcluster percentage distribution at different time points in the rat anti-Thy1.1 MsPGN model. (**E**) Bar plot showing the percentage of MC subclusters at different time points. (**F**) Dot plot showing the expression of representative marker genes of MC subclusters. (**G**) The trajectories of MC subclusters. (**H**) The expression of Cxcl12, Cxcl16, and Csf1 in MC subclusters at different time points in the rat anti-Thy1.1 MsPGN model. (**I**) Bubble plot showing significant ligand-receptor pairs for immune cell and MC subclusters in the rat anti-Thy1.1 MsPGN model. Two-tailed *t* test. (**J**) Immunostaining showing *Cxcl12* expression in the anti-Thy1.1 model at 0, 1, 3, 5, 7, and 10 days, at original magnification, ×400. (**K**) PAS staining of kidney tissue (at original magnification, ×400) and proteinuria levels in the control, Thy1.1, and Thy1.1+AMD3100 groups (*n* = 7); *** *P* < 0.001, **** *P* < 0.0001 versus control, ^###^, *P* < 0.001; ^####^, *P* < 0.0001 Thy1.1 versus Thy1.1+AMD3100. (**L**) Levels of Tgf-β, Pdgfrb, Et-1, and C3 in the glomeruli of each group detected by Western blotting. Data are expressed as mean ± SD; 1-way ANOVA was used for comparisons of 3 or more groups.

Having shown the effects of CXCL12 secreted by MCs on macrophage function in vitro, we went on to test the potential role of the CXCL12/CXCR4/C3 pathway in immune injury and renal function in vivo. First, we generated the rat anti-Thy1.1 MsPGN model (Supplemental Figure 8) and conducted a single-cell survey to decipher the cellular characteristics of the anti-Thy1 MsPGN model (Figure 5A). Analysis of the scRNA-Seq data at 0 days (sham), 3 days (mesangial lysis stages), and 7 days (mesangial proliferation stage) revealed 51,059 cells and 11 main cell types (Figure 5, A–C, and Supplemental Table 4); the percentage of T cells, B cells, and Neu increased greatly in 3 days, and the percentage of MCs and Macs was higher in 7 days (Figure 5C), which supported that MsPGN was characterized by abundant immune cell infiltration and proliferation of MCs. Detailed analysis of MCs revealed 3 subtypes: proliferative MCs (marked by Mki67, Pdgfrb), MCs (marked by Pdgfrb), and inflammatory MCs (iMCs, marked by Pdgfrb, Cxcl12, Csf1, Il34, Cxcl16) according to representative marker genes (Figure 5, D–F, and Supplemental Table 5), and iMCs originated from the differentiation of the proliferative MCs by pseudotime trajectory analysis (Figure 5G). Interestingly, the percentage of iMCs (Figure 5E) as well as the expression of Cxcl12, Il34, and Cxcl16 increased alongside the MsPGN progression process (Figure 5H). Further cellular crosstalk analysis revealed that Cxcl12/Cxcr4 and Il34/Csf1 receptor (Csf1r) played an important role in the interaction of MCs and immune cells, which supported the scRNA-Seq findings in human IgAN and ddY mouse IgAN (Figure 5I). Immunofluorescence analysis revealed that Cxcl12 expression was substantially elevated during the mesangial lysis stages followed by a marked decline in the mesangial proliferation and recovery stages (Figure 5J). Analysis of pathological periodic acid–Schiff (PAS) staining showed that AMD3100 treatment (Thy1.1+AMD3100) markedly reduced the number of cells per glomerular cross section, indicating mesangial proliferation was inhibited; proteinuria levels were also reduced in the Thy1.1+AMD3100 group, compared with the model group (Thy1.1+NS) (Figure 5K). Western blotting further showed that the levels of indicators of MC injury such as Tgf-β, Pdgfrb, and Et-1, as well as C3, which can cause damage to MCs in the glomeruli, were increased in the nephritis model group (Figure 5L). Following treatment with AMD3100, the expression of all the above indicators showed a downward trend. Thus, Cxcl12 expression was highly correlated with the degree of pathological injury. These findings in rat MsPGN along with the proteomic results in human IgAN samples demonstrated that Cxcl12 is an important indicator of IgAN progression.

### Alleviation of immune injury and decline of renal function by blockade of CXCL12/CXCR4/C3.

**Figure 6:**
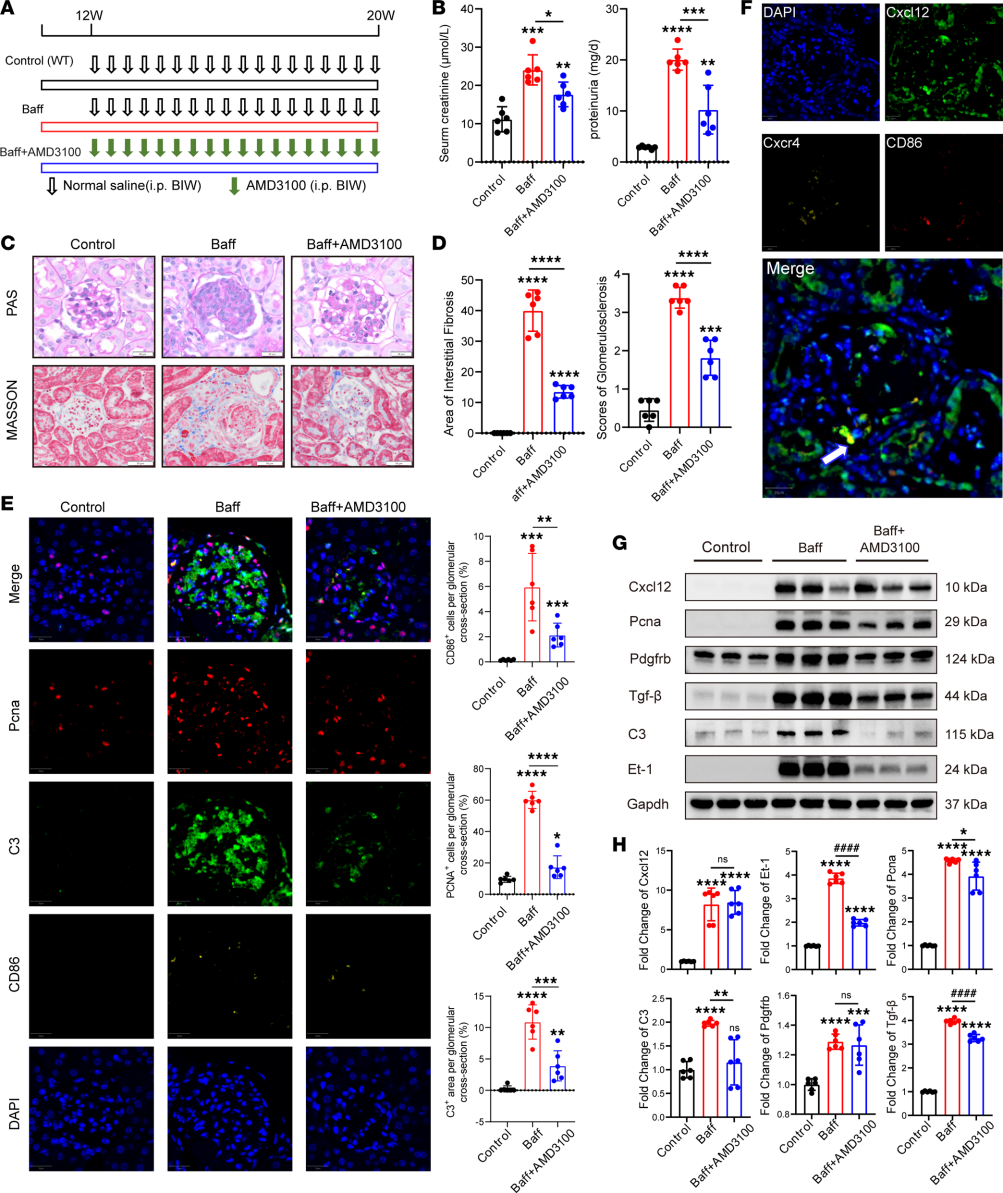
Effects of Cxcr4 blockade on kidney in the BAFF IgAN model. (**A**) Schedule of treatments in the in vivo intervention experiment. Green arrows indicate AMD3100 injection; black-edged arrows indicate normal saline injection. BIW, twice a week; i.p., intraperitoneal injection. (**B**) Serum creatinine and proteinuria levels in the control, BAFF, and BAFF+AMD3100 groups (*n* = 6). (**C** and **D**) PAS staining and Masson staining of kidney tissue (at original magnification, ×400) in the control, BAFF, and BAFF+AMD3100 groups (*n* = 6). (**E**) Detection of PCNA, CD86, and C3 in the glomeruli of each group by immunofluorescence (at original magnification, ×400) and semiquantitative analysis of expression levels (*n* = 6). (**F**) Detection of CD86, Cxcl12, and Cxcr4 in the glomeruli by immunofluorescence (at original magnification, ×400). The white arrow indicates that the macrophage that expressed CD86 and Cxcr4 resided near MCs with high expression of Cxcl12 in glomeruli. (**G** and **H**) Levels of Cxcl12, Pcna, Tgf-β, Pdgfrb, Et-1, and C3 in the glomeruli of each group detected by Western blotting, with semiquantitative analysis (*n* = 6). *** *P* < 0.001, **** *P* < 0.0001 versus control, * *P* < 0.05, ** *P* < 0.01, ^####^ *P* < 0.0001 BAFF versus BAFF+AMD3100. Data are expressed as mean ± SD (*n* = 6); 1-way ANOVA was used for comparisons of 3 or more groups.

Next, we performed Cxcr4 blockade experiments (Figure 6A) in the mouse IgA glomerulonephritis model (16, 19), a transgenic mouse that expresses excessive BAFF, which exhibits features of autoimmune disease, including B cell hyperplasia and hypergammaglobulinemia, and develops IgAN with circulating immune complexes and immunoglobulin deposition in the kidney (Supplemental Figure 9). Analysis of serum creatinine and proteinuria showed that the BAFF group had significant decline in renal function while treatment with AMD3100 could alleviate this trend (Figure 6B). The pathological PAS staining and Masson staining showed that AMD3100 treatment (BAFF+AMD3100) significantly reduced the glomerulosclerosis and fibrosis compared with the model group (BAFF) (Figure 6, C and D). Immunofluorescence analysis of tissue samples with AMD3100 treatment (BAFF+AMD3100) also showed that the infiltration of CD86^+^ macrophages, degree of C3 deposition, and proliferation of MCs were significantly reduced, compared with the model group (Figure 6E). Immunofluorescence colocalization showed that C3 deposition was greater at sites of macrophage infiltration. We also found that CD86^+^ macrophages also expressed Cxcr4 and resided on the Cxcl12^hi^ region (Figure 6F). Western blotting further showed that the levels of MC proliferation indicator Pcna and indicators of MC injury such as Tgf-β, Pdgfrb, and Et-1, as well as C3, which can cause damage to MCs in the glomeruli, were increased in the BAFF group. Additionally, we found that indicators of tubular injury and fibrosis such as α-SMA, FN1, and VIM were also increased in the BAFF group (Supplemental Figure 10). Following treatment with AMD3100, the expression of all the above indicators showed a downward trend (Figure 6, G and H, and Supplemental Figure 10). These results indicated that inhibiting the Cxcl12 receptor led to reduced numbers of macrophages, suppressed the deposition of complement in glomeruli, and alleviated MC injury and proliferation. Thus, blocking Cxcl12/Cxcr4/C3 signaling may alleviate the degree of inflammatory injury, fibrosis, and decline of renal function in IgAN models.

## Discussion

**Figure 7:**
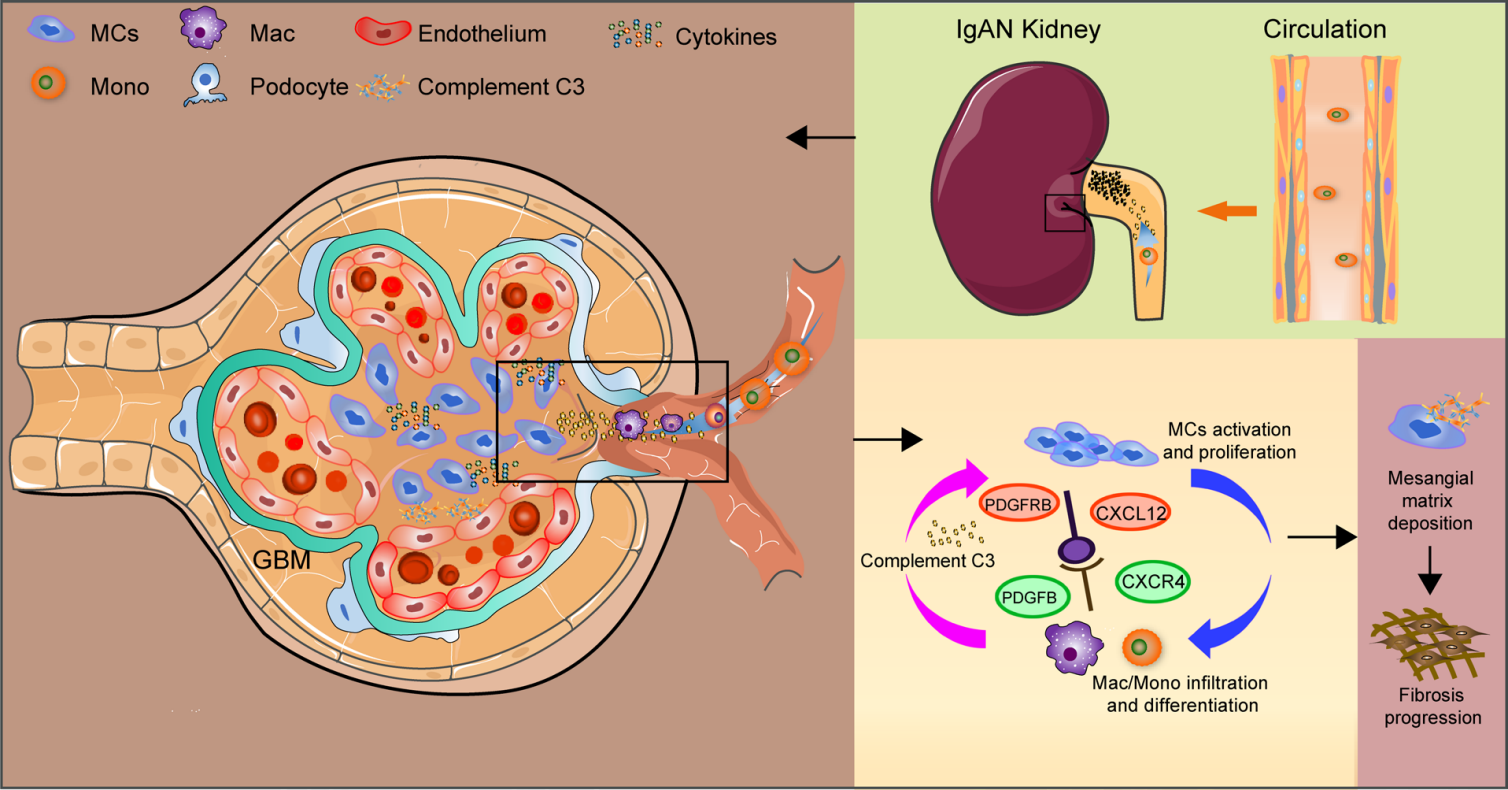
A simple model for the immunopathogenesis mechanism associated with IgAN progression, indicating CXCL12/CXCR4/C3 may be potential targets for IgAN precision therapy. GBM, glomerular basement membrane.

In most human glomerulopathies, including IgAN, abnormal MC activation and proliferation and podocyte injury and loss correlate highly with the loss of kidney function. Cross-species characterization of the key cell types and molecules in the human and mouse IgAN microenvironments is necessary to aid in the identification of precise targets for IgAN therapy. In this study, we combined scRNA-Seq, bulk RNA-Seq, and proteomic approaches to survey the major cell players and intercellular crosstalk–associated molecules in IgAN that are conserved across species. Our analyses demonstrated a highly heterogenous IgAN microenvironment at the single-cell level, supporting the role of infiltrating monocytes/macrophages and abnormally activated/proliferating MCs in IgAN progression (Figure 7). Moreover, our results highlighted the roles of the CXCL12/CXCR4 axis and complement C3 protein in intercellular crosstalk between MCs and macrophages and IgAN progression. Overall, these findings shed light on the conserved pathological mechanisms associated with IgAN.

In this study, DEG analysis revealed the conserved expression of cell-specific genes such as *PDGFRB*, *CXCL12*, and *IL34* in MCs from humans and mice. To identify conserved features, this study used the ddY mouse, the animal model used most frequently to study IgAN. In addition, we conducted an unbiased scRNA-Seq survey in an MsPGN model and uncovered an iMC phenotype that expressed Cxcl12, Pdgfrb, Il34, Cxcl16, which correlated with the MsPGN injury progression. To the best of our knowledge, our study is the first to decipher the pathological interactions between MCs and macrophages at single-cell resolution and highlight the importance of the CXCL12/CXCR4/C3 pathway in IgAN progression. Macrophages also expressed high levels of *PDGFB*, *TNF*, and *C3* — reported to be important inducers of MC activation and proliferation — and acted on MCs mainly via the PDGFB/PDGFRB and C3/ITGAX/ITGAM pathways. Moreover, high levels of CXCL12 in IgAN tissues correlated with IgAN progression and worse prognosis in human IgAN. CXCL12 (also known as SDF1) is a chemokine; this class of low–molecular weight cytokines comprises key mediators that promote cell migration during routine immune monitoring, inflammation, and development and regulate the entry of immune cells into target tissues by binding to their corresponding receptors (20, 21). Binding of CXCL12 to its receptor, CXCR4, facilitates the homing of immune cells to secondary lymphatic organs and regulates cell migration in lymph nodes (22). CXCL12 is a key mediator of repair in many disease models, such as islet β cell loss in type 1 diabetes (23), intravascular injury (24), vascular obstruction (25), and ischemic acute renal failure (26). However, in chronic disease conditions, CXCL12 is often characterized by maladaptive damage response mechanisms, which can promote disease progression and even lead to organ failure. For example, CXCL12 can mediate the recruitment of fibrocytes, which exacerbates bleomycin-induced pulmonary fibrosis (27). Nephrotoxic serum glomerulonephritis specifically induces glomerular CXCL12 expression, while transgenic overexpression of CXCR4 induces podocyte proliferation and glomerular crescent formation in mice (28). Moreover, in NZB/NZW mouse lupus immune complex nephritis, blocking CXCL12 can prevent glomerulonephritis by decreasing autoantibody production and T cell recruitment by glomeruli (29).

Our in vivo experiments using the anti-Thy1.1 nephritis model and BAFF mouse IgAN model revealed that complement C3 was associated with pathological injury and progression of the lesions. Although circulating C3 is produced by the liver, extrahepatic production has been observed in other specialized cells, including mast cells, fibroblasts, smooth muscle cells, and macrophages. Plasma complement C3 levels can be used as a diagnostic and prognostic indicator of IgAN, illustrating the close relationship between complement C3 and IgAN. The rat anti-Thy1.1 nephritis model simulates human MsPGN as well as the local pathological changes of IgAN (30, 31), including the proliferation of MCs and extracellular matrix deposition (32). Our findings support previous evidence that the anti-Thy1.1 model is complement dependent (33). In the glomeruli of patients with IgAN, complement C3 is usually deposited in areas where IgA antibodies and other immune molecules are deposited, forming the basis of an inflammatory response and glomerular damage. Nonetheless, to date, the origin of C3 and its mechanism of deposition in the glomerular mesangium in IgAN have been unclear. In this study, we showed that macrophages in IgAN samples expressed high levels of CXCR4 and C3, while pseudotime-based differentiation trajectory analysis indicated that these kidney macrophages derived from the infiltration and differentiation of blood monocytes. Hence, we speculate that in IgAN, CXCL12-expressing MCs may recruit blood monocytes/macrophages into tissue via the CXCL12/CXCR4 pathway; secretion of high levels of C3, TNF, and PDGFB by macrophages may then promote MC activation and C3 deposition. Consistent with these findings, previous studies have suggested that glomerulosclerosis can be prevented by blocking the recruitment of macrophages to glomeruli (34). Our hypothesis was validated by in vitro coculture assays showing that the addition of exogenous CXCL12 or overexpression of *Cxcl12* in MCs enhanced the migratory ability of macrophages. Importantly, specific blockade of the Cxcl12 receptor Cxcr4 in vivo using AMD3100 substantially reduced the infiltration of macrophages, expression of C3, and level of pathological injury. In previous studies, AMD3100 also reduced the expression of PDGFB in macrophages (35). PDGFB is an important cytokine that promotes mesangial cell proliferation whereas its receptor, PDGFR, is mainly expressed in MCs and other renal stromal cells, including interstitial fibroblasts, perivascular cells, and paraglomerular cells, in glomeruli (36). Its activation and amplification often reflect the pathological proliferation and fibrosis of the kidney (37).

Our research has some limitations. Although we used the rat anti-Thy1.1 nephritis model to validate the results of the bioinformatics analysis, this model does not accurately simulate the pathogenesis of IgAN. Nonetheless, this is not necessarily a disadvantage as our use of the model focused on the mechanism of local immunopathological damage to the kidney after the occurrence of IgAN, rather than pathogenesis. The rat anti-Thy1.1 nephritis model can be used to show pathological changes after immune complex or antibody deposition in glomeruli; moreover, because of the self-limited nature of this model, we were able to observe the key cytokines and molecular features involved in the self-repair process of MCs in glomeruli. Thus, the use of the anti-Thy1.1 nephritis model provided valuable insights into glomerular repair after IgAN as well as potential therapeutic targets.

In conclusion, we have presented the what we believe is the first comprehensive cross-species single-cell transcriptomic analysis in IgAN, revealing the pathological cell types and intercellular crosstalk involved in IgAN initiation and progression. By elucidating the critical progression-associated cell players and pathways conserved between humans and mice, our results provide a solid foundation for future studies to develop effective strategies for IgAN treatment.

## Methods

### Sex as a biological variable.

For the human IgAN proteomic profiling study, 26 women and 23 men were enrolled. The detailed information can be seen in Supplemental Table 3. Sex was not considered as a biological variable in animal studies.

### Processing of scRNA-Seq data and cell type determination.

Gene expression matrixes for the sequenced rat MsPGN, human IgAN, and mouse ddY samples were produced using the Cell Ranger (v5, 10x Genomics) count function via the STAR algorithm by aligning raw sequencing FASTQ files to the mRatBN7.2, GRH38, and mm10 reference genomes, respectively. Seurat (38) R package (version 4.0.0) was employed to perform subsequent data analysis, including normalization, scaling, principal component analysis (PCA), UMAP dimension reduction, and visualization of gene expression. We filtered low-quality cells that had <2,001 unique molecular identifiers (UMIs), >6,000 or <501 expressed genes, or >20% of UMIs derived from the mitochondrial genome. We removed potential cell doublets using the DoubletFinder (39) R package as the dropout effect of 10x Genomics data and used the SeuratWrappers package to integrate single-cell transcriptome expression using the RunFastMNN function. Then, we selected highly variable genes for PCA; the top 30 significant principal components were selected for UMAP dimension reduction and visualization of gene expression. Cell types were annotated according to known canonical marker genes and DEGs calculated using the FindAllMarker function with the default parameters provided by Seurat.

### Trajectory analysis.

To illustrate potential cellular differentiation routes and dissect the origins of infiltrating macrophages and monocytes, the top 150 signature genes were calculated using the differentialGeneTest function provided by the Monocle algorithm (40). Cell differentiation trajectories were inferred using default Monocle parameters after dimension reduction and cell ordering. Then, the DDRTree function was used for dimensionality reduction, and the plot_cell_trajectory function was used for visualization.

### Pathway analysis and cell subtype deconvolution in bulk RNA-Seq samples.

We used gene set variation analysis (GSVA) (41) to assess pathway enrichment in IgAN and normal control samples using the Hallmark gene sets provided by the Molecular Signatures Database; this analysis was performed via a linear model offered by the limma package. We employed the GSVA package to assess the IgAN bulk RNA-Seq data for the relative abundance of the cell subtypes identified by scRNA-Seq.

### Intercellular crosstalk analysis.

To explore intercellular crosstalk networks, we assessed ligand-receptor distribution and expression levels in infiltrating immune cells and MCs via a standard pipeline implemented in R using the CellChat (42) R package, as previously reported. First, we established a potential ligand-receptor interaction list by projecting the human gene expression data onto the protein-protein interaction network and identifying overexpressed ligand-receptor pairs. To distinguish the biologically significant cell-cell communication pathways, probability values for each interaction were calculated by performing permutation tests. We selected receptors and ligands expressed in more than 10% of the cells in a specific cluster for subsequent analysis. Interaction pairs with ligands belonging to the complement, PDGFB, and CXCL families were selected for the evaluation of intercellular crosstalk between distinct MCs and infiltrating macrophages. Inferred intercellular communication networks for each ligand-receptor pair and signaling pathway were summarized and visualized using circle plots and heatmaps.

### Human IgAN proteomic analysis and clinical prognosis analysis.

The protein expression data of 49 IgAN samples can be downloaded from our previous study in ProteomeXchange Consortium via the iProX partner repository (PXD032710). The primary outcome of human patients with IgAN was defined as an eGFR decline more than 30% in the follow-up time, which classified the 49 patients into progression group and nonprogression group. Significantly changed proteins in human IgAN nonprogression group or progression group were determined using unpaired Welch’s *t* test implemented in statistical software environment R (version 3.3.2) and screened by heatmap and volcano plot analysis using R package ComplexHeatmap and ggplot2, respectively. Proteins with a *P* < 0.05 and a FC > 0.5 between the nonprogression group and the progression group were considered significant. For survival analysis, the Cox proportional hazards model was used. The hazard ratios of protein signature were calculated using a single-factor Cox regression model and visualized by forest plot.

### Mice.

BAFF-Tg mice and WT mice (20 ± 2 g, male, purchased from Beijing GemPharmatech Co. Ltd.) were adaptively fed using a 12-hour light/12-hour dark cycle with freely accessible diet and water. AMD3100, a recognized inhibitor of Cxcr4 (43), was administered by intraperitoneal injection at a dose of 2 mg/kg (in 0.5 mL normal saline). The BAFF+AMD3100 group received an injection of AMD3100 (ApexBio) twice a week from 12 to 20 weeks, and then samples were collected. The control group and BAFF group were injected with vehicle without AMD3100, and the other operations were the same as the BAFF+AMD3100 group.

### Establishment of the anti-Thy1.1 nephritis model.

Wild-type Wistar rats (200 ± 20 g, male, purchased from Beijing SPF Biotechnology Co., LTD.) were adaptively fed using a 12-hour light/12-hour dark cycle for 1 week with freely accessible diet and water. The anti-Thy1.1 nephritis model was established by injecting anti-Thy1.1 antibody (provided and produced by State Key Laboratory of Kidney Diseases) via the tail vein of the rats at a concentration of 2.5 mg/kg, as previously described (25, 26). The control group (0 day) was injected with the same volume of normal saline.

### Biochemical tests.

Serum creatinine and concentrations were tested using BioAssay kits (BioAssay Systems). Urine protein concentrations were measured using the Thomas brilliant blue method (CBB method), and total urine protein was calculated based on the 24-hour urine volume.

### Pathological staining.

The samples were dehydrated, permeated with paraffin wax, embedded, sectioned (2 μm), and then subjected to histological staining with PAS or Masson according to standard protocols. Then, the sections were dehydrated and sealed with resin.

### Immunofluorescence.

For immunofluorescence, the paraffin sections were dewaxed, and antigen retrieval was carried out before staining by microwaving the samples in citrate buffer solution. Akoya Biosciences Opal mIHC (TSA) was used according to the instructions. The primary antibodies were anti-Cxcl12 (1:200, ab9797, Abcam), anti-Cxcl12 (1:50, sc-74271, Santa Cruz Biotechnology), anti-Cxcr4 (1:50, ab181020, Abcam), anti-PCNA (1:50, ab29, Abcam), anti-C3 (1:50, pt21337, Proteintech), anti-CD86 (1: 50, ab119857, Abcam) and anti-PDGFRβ (1:50, ab69506, Abcam).

### Western blotting.

Proteins were extracted from tissues and cells using RIPA lysis buffer. Kidney tissue was ground and the glomeruli and renal tubules were then isolated by sieving and used for analyses. The samples were electrophoresed, transferred onto membranes, and blocked; the membranes were then incubated with primary and secondary antibodies, and the Bio-Rad ChemiDoc system was used for luminescence. The primary antibodies were anti-PCNA (1:50, ab29, Abcam), anti-C3 (1:1,000, 21337, Proteintech), anti-Cxcl12 (1:200, sc-74271, Santa Cruz Biotechnology), anti-GAPDH (1:10,000, 60004, Proteintech), anti–TGF-β1 (1:500, 21898, Proteintech), anti-PDGFRβ (1:1,000, 13449, Proteintech), and anti–endothelin 1 (1:1,000, ab2786, Abcam). The secondary antibodies were HRP-labeled goat anti-rabbit (1:1,000, A0208, Beyotime) and HRP-labeled goat anti-mouse (1:1,000, A0216, Beyotime).

### Quantitative reverse transcription PCR.

We used TRIzol (Invitrogen) to extract RNA from tissues and samples according to the manufacturer’s instructions. The RNA was then reverse-transcribed into cDNA using ProtoScript II First Strand cDNA Synthesis Kit (New England Biolabs), and quantitative reverse transcription PCR was performed using SYBR Select Master Mix (Applied Biosystems) according to the manufacturer’s instructions.

### Cell culture.

Murine glomerular MC lines (SV40 MES 13, CRL-1927) and macrophage cell lines (RAW 264.7, TIB-71) were purchased from the American Type Culture Collection. The cells were cultured in 4.5 g/L glucose DMEM in a humidified environment at 37°C with 5% CO_2_ and 21% O_2_.

### Establishment of Cxcl12-knockdown and -overexpression MC models.

siRNA was used to knock down Cxcl12, and the plasmid containing Cxcl12 cDNA was used to overexpress Cxcl12 in MCs. Cxcl12 siRNA and the overexpression plasmid were purchased from GenePharma Co., Ltd, and the sequence of the plasmid is ATGGACGCCAAGGTCGTCGCCGTGCTGGCCCTGGTGCTGGCCGCGCTCTGCATCAGTGACGGTAAACCAGT CAGCCTGAGCTACCGATGCCCCTGCCGGTTCTTCGAGAGCCACATCGCCAGAGCCAACGTCAAGCATCTG AAAATCCTCAACACTCCAAACTGTGCCCTTCAGATTGTTGCACGGCTGAAGAACAACAACAGACAAGTGTGCATTGACCCGAAATTAAAGTGGAT CCAAGAGTACCTGGAGAAAGCTTTAAACAAGTAA. siRNA was transfected using Lipofectamine RNAiMAX Reagent (Invitrogen), and the plasmid was transfected by Lipofectamine 3000 Reagent (Invitrogen).

### Coculture systems.

Coculture systems were established using 24-well Transwell plates. For the macrophage migration assay, macrophages were inoculated into the Transwells, and MCs were inoculated into the plate wells. When the MC fusion was about 60%–70%, MCs were treated with Cxcl12 knockdown/overexpression, and only transfection reagents were added to the control group. After coculture for 24 hours, macrophages were stained with crystal violet for 20 minutes, and the number of cells of 9 fields of vision under the microscope (Olympus, DP72) in the Transwell chamber was counted and analyzed. For the macrophage proliferation assay, MCs were inoculated into the Transwells, and macrophages were inoculated into the plate wells. After being cocultured, macrophages were subjected to EdU assay.

### EdU assay.

The EdU Staining Proliferation Kit (iFluor 488, ab219801, Abcam) was used for EdU assays according to the manufacturer’s instructions.

### Wound-healing assay.

Macrophages were incubated in 6-well plates. When cell confluence reached around 70%, sterilized 200 μL pipette tips were used to draw 3 parallel lines per well, intersecting vertically at the marker line. Following the addition of Cxcl12 (100 ng/mL, PeproTech), the cells were immediately observed under the microscope and photographed (0 hour). Then, the cells were incubated and photographed at 24 and 48 hours. The images were processed and analyzed using ImageJ software (NIH) to calculate the degree of cell migration between delineations.

### Statistics.

Data from more than 3 independent experiments were represented as scatterplots with bars and analyzed by unpaired 2-tailed *t* test or 1-way ANOVA using SPSS Statistics 23 software. Differences were considered statistically significant at *P* < 0.05.

### Study approval.

The animal care and experimental procedures were approved by the ethics committees for animal experimentation at the Chinese PLA General Hospital (No. 2022-X18-30). The human IgAN biopsies for proteomics profiling and immunostaining were collected from Chinese PLA General Hospital, with the approval of the Research Ethics Committee, with ethics committee approval number S2015-061-01.

### Data availability.

The rat anti-Thy1 MsPGN model scRNA-Seq data required to reproduce the analysis and figures have been deposited on Zenodo’s data set website (https://zenodo.org/records/8045416). Additional data sets analyzed in this study are available from the NCBI GEO repository under the accession numbers GSE127136 (9) GSE166793 (44), and GSE93798 (17) and ProteomeXchange Consortium via the iProX partner repository (PXD032710). Values for all data points found in graphs are in the Supporting Data Values file.

## Author contributions

GC, Xiangmei Chen, and MZ conceived and designed the experiments. TW, Xizhao Chen, and LC conducted the experiments. MZ performed the data analysis. WS, LL, ZY, CZ, and YD provided valuable advice and were responsible for research supervision, coordination, and strategy. Xizhao Chen and MZ drafted the manuscript. GC and Xiangmei Chen reviewed and edited the manuscript. All authors read and approved the final manuscript.

## Supplementary Material

Supplemental data

Unedited blot and gel images

Supplemental tables 1-5

Supporting data values

## Version 1 05/08/2024

Electronic publication
