# Supplementary material for: Cross-species single-cell analysis uncovers the immunopathological mechanisms associated with IgA nephropathy progression
Source: JCI Insight. 2024 May 8;9(9):e173651. doi: 10.1172/jci.insight.173651 (PMC11141938; doi:10.1172/jci.insight.173651)
Supplement: Supplemental data [file jciinsight-9-173651-s027.pdf]

Supplemental figures and legends

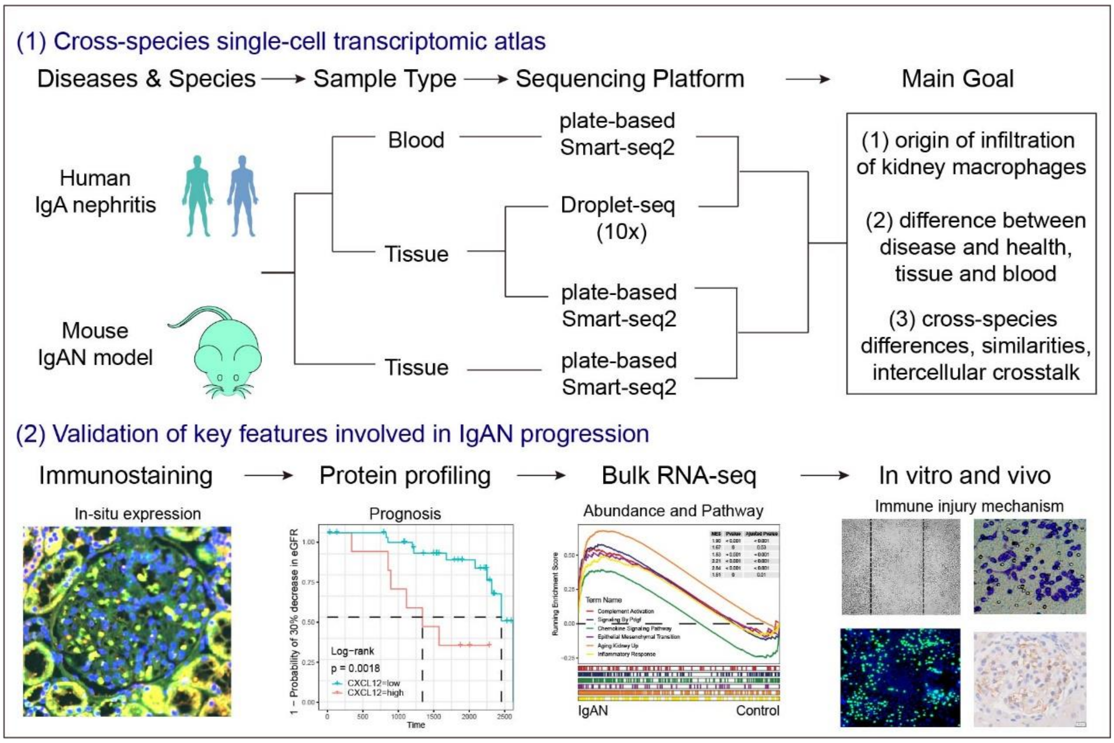

Figure S1: Study design, data acquisition and main goals

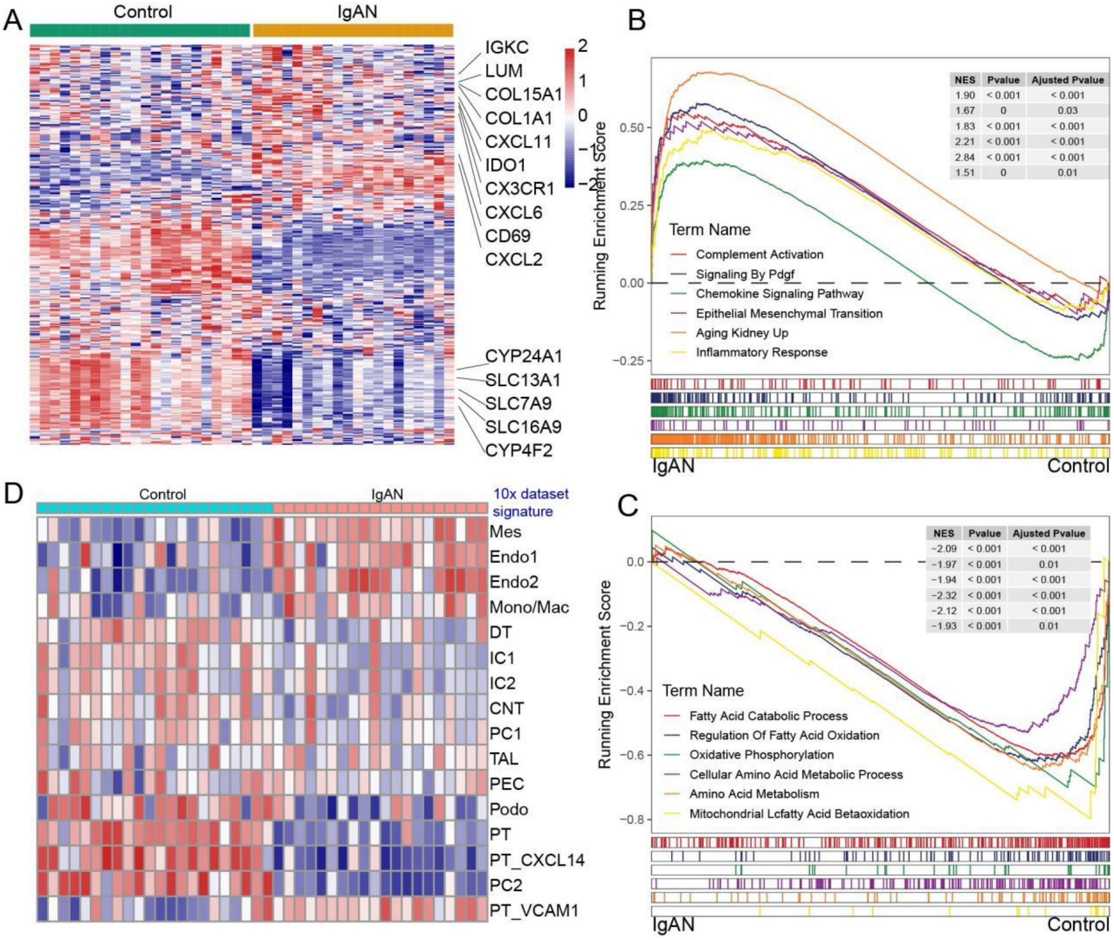

Figure S2: Validation of IgAN scRNA-seq findings using bulk RNA-seq dataset. (A) The DEGs of human IgAN samples and healthy control kidney samples. (B-C) The upregulated pathways (B) and downregulated pathways in IgAN compared to healthy control kidney samples based on the top 50 DEGs.

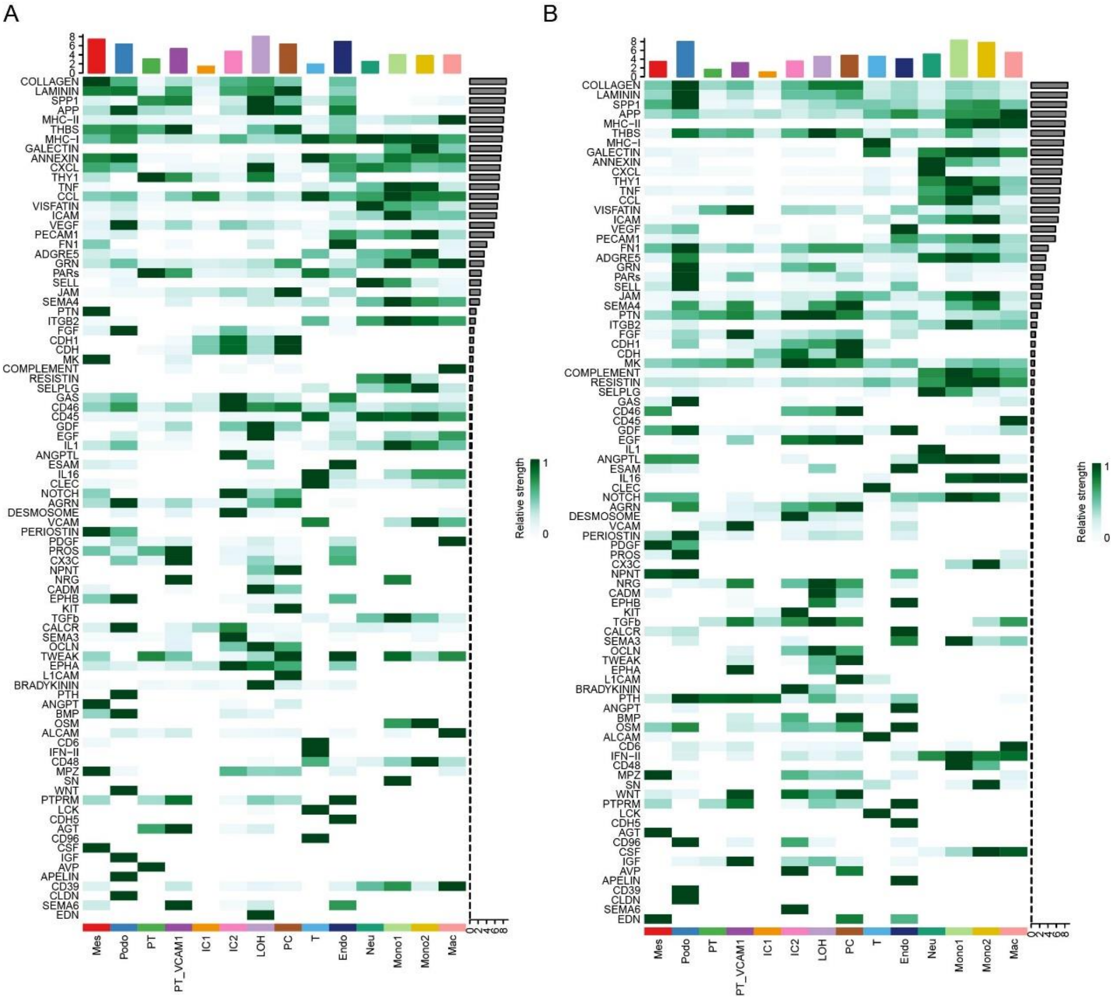

Figure S3: Intercellular crosstalk analysis showing the outcoming (A) and incoming (B) signaling pathways enriched in human IgAN ecosystem.

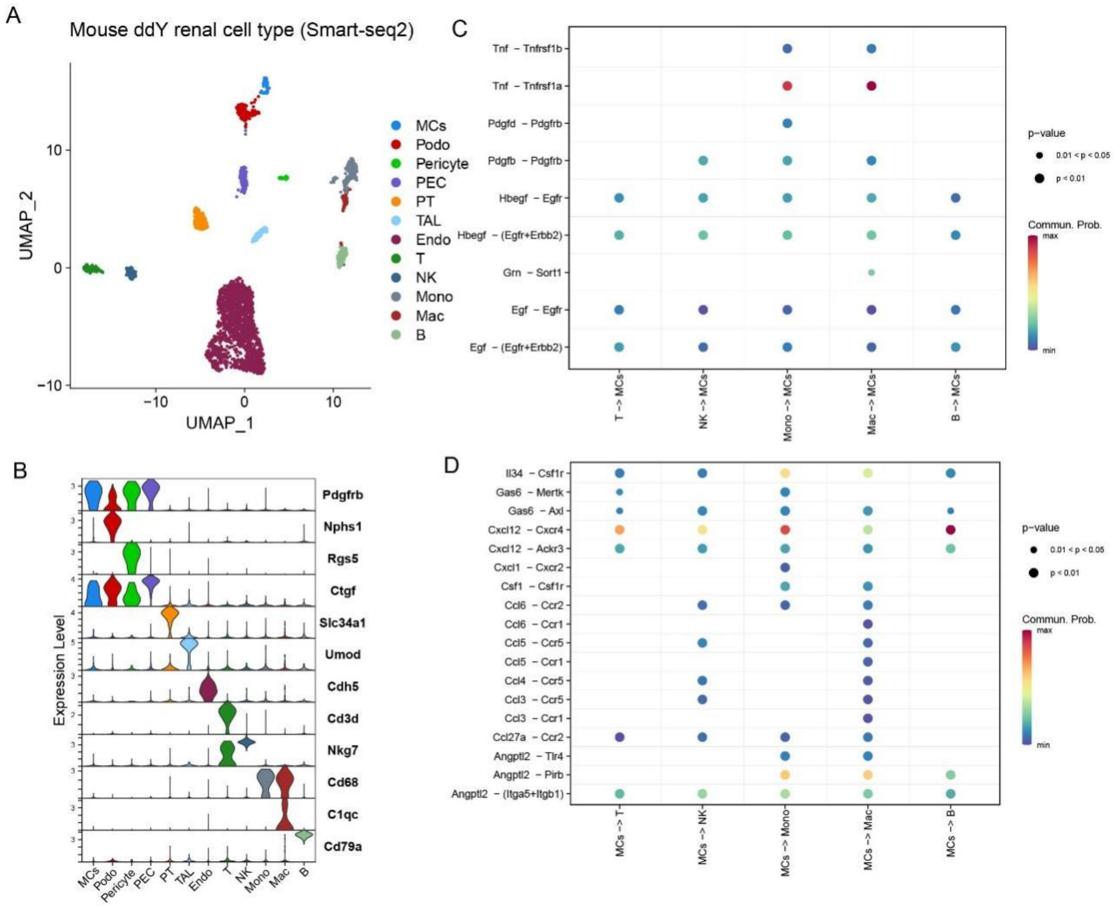

Figure S4: Intercellular crosstalk analysis showing the enriched ligand-receptors in mouse ddY IgAN ecosystem.

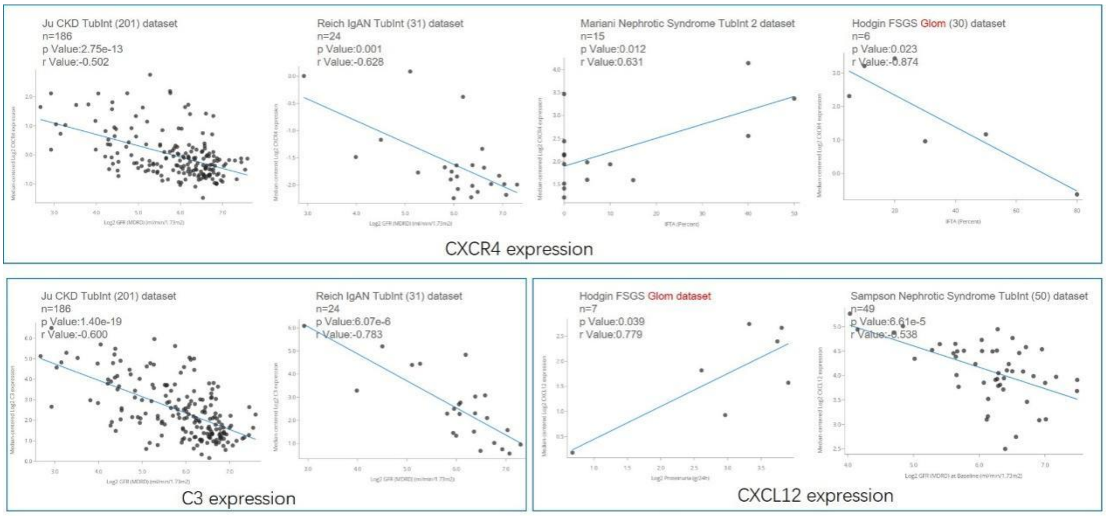

Figure S5: The correlation of the expression level of CXCL12-CXCR4-C3 and the eGFR, interstitial fibrosis and tubular atrophy (IFTA) score, and proteinuria in CKD including IgAN patients from the Nephroseq datset (https://www.nephroseq.org/resource).

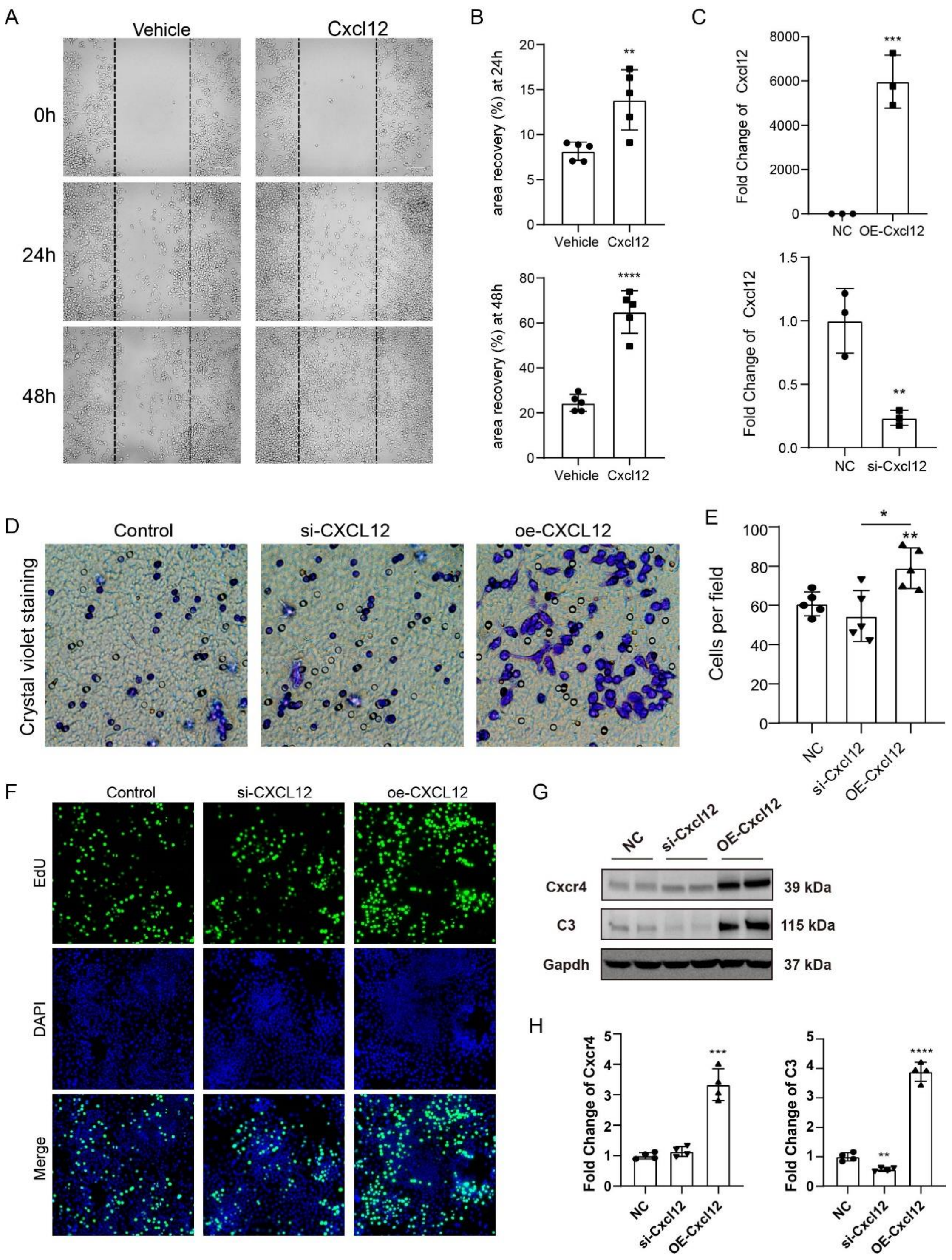

Figure S6 Effects of Cxcl12 on macrophages in vitro. (**A-B**) Wound healing assays showing the degree of macrophage migration in the control group and Cxcl12-treated group at 24 h and 48 h (n = 5); ** $p < 0.01$, **** $p < 0.0001$ Cxcl12 versus vehicle. (**C**) Knockdown and overexpression of *Cxcl12* in mesangial cells verified by qRT-PCR. (**D-E**) Crystal violet staining showing the migration of macrophages following co-culture with mesangial cells with knockdown (si-Cxcl12) or overexpression (OE-Cxcl12) of *Cxcl12* (n = 5). * $p < 0.05$, si-Cxcl12 versus OE-Cxcl12; ** p <

0.01 OE-Cxcl12 versus control. (**F**) EdU assay carried out on macrophages co-cultured with mesangial cells with knockdown or overexpression of *Cxcl12*, at original magnification ×200. (**G-H**) Western blots showing the levels of Cxcr4 and C3 proteins in macrophages co-cultured with mesangial cells with knockdown or overexpression of *Cxcl12* (n = 4). ** $p < 0.01$, *** $p < 0.001$, **** $p < 0.0001$ versus NC.

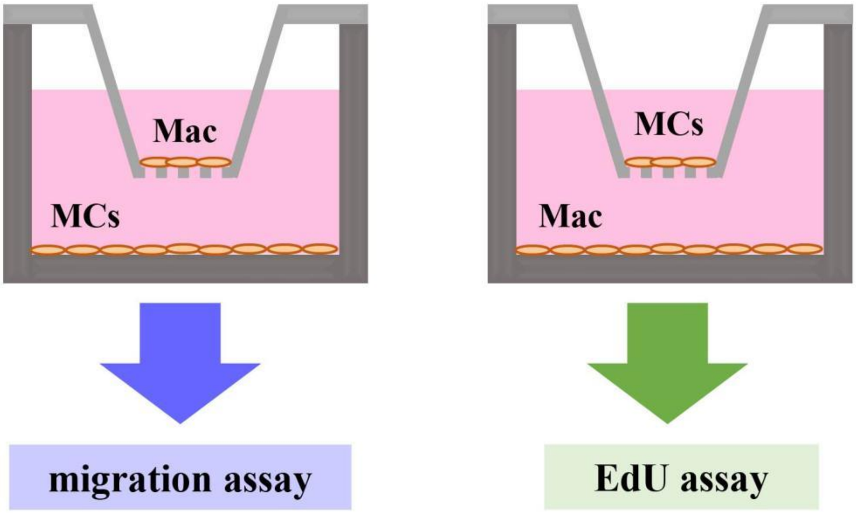

Figure S7: A brief model showing the migration assay and Edu assay in transwell couture system.

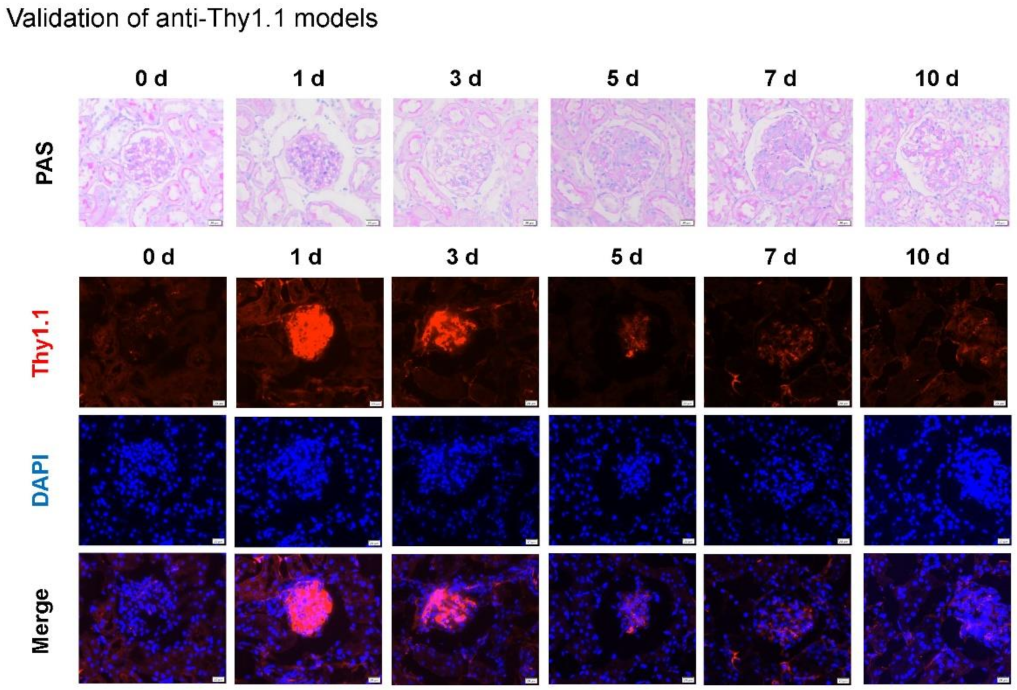

Figure S8: Immunostaining experiment showing the anti-Thy1.1 MsPGN model, across different time points.

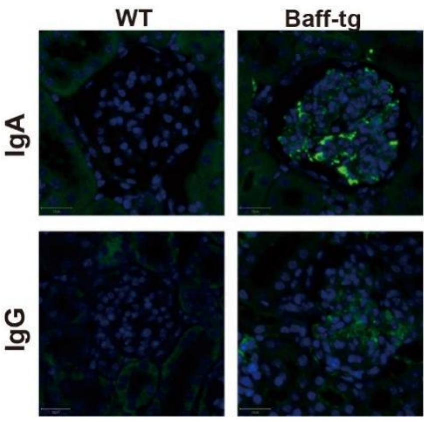

Figure S9: Immunostaining experiment showing the expression of IgA and IgE in mouse BAFF IgAN model and control.

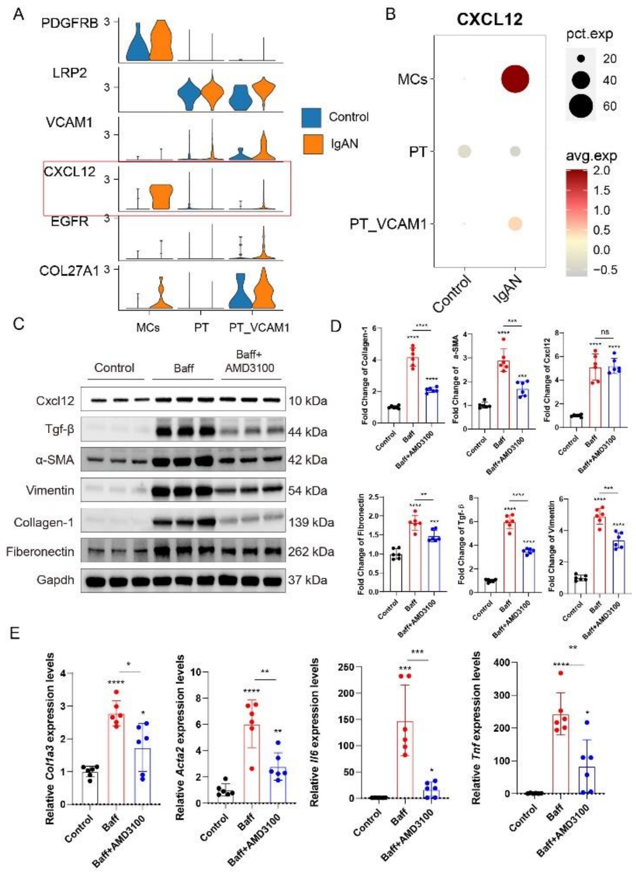

Figure S10: The role of CXCL12 in the renal tubules and interstitium. (**A-B**) The Violin diagram and dotplot showing the expression of CXCL12 in the MCs, PT and PT_VCAM1 (**C**) The results of Western Blots showed that the expression of Cxcl12 were upregulated in the BAFF and BAFF+AMD3100 groups. (D) The markers of renal tubulointerstitial fibrosis α-SMA, Collagen-1, Vimentin and Fibronectin in BAFF model were significantly higher than those in control group and BAFF+AMD3100 group. (**E**) qRT-PCR showed that the expression of the markers of renal tubulointerstitial fibrosis α-SMA and Fibronectin and inflammatory markers Il6 and TNF-α in BAFF model were significantly higher than those in control group and BAFF+AMD3100 group.
