# Supplementary material for: Cross-species single-cell analysis uncovers the immunopathological mechanisms associated with IgA nephropathy progression
Source: JCI Insight. 2024 May 8;9(9):e173651. doi: 10.1172/jci.insight.173651 (PMC11141938; doi:10.1172/jci.insight.173651)

Figure 5

Gapdh

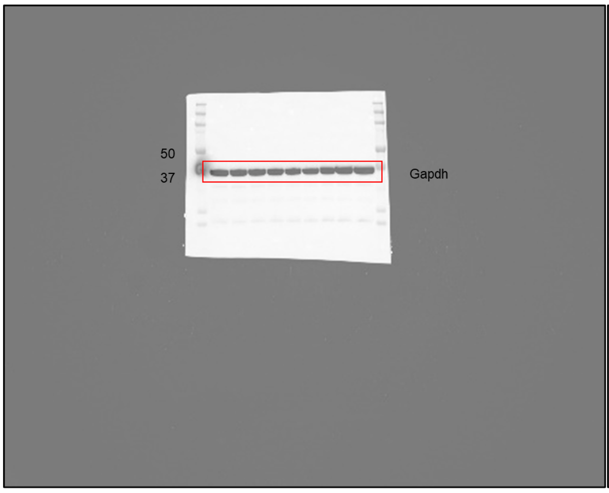

Tgf-β

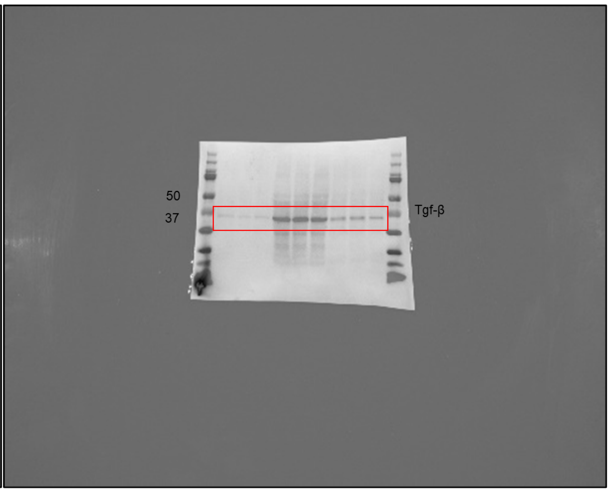

C3

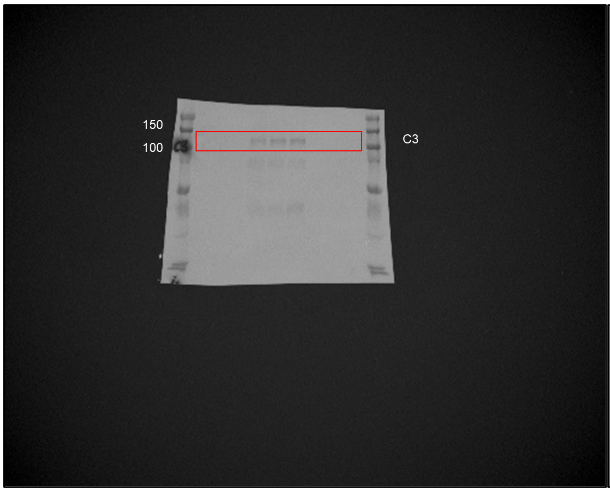

Pdgfrb

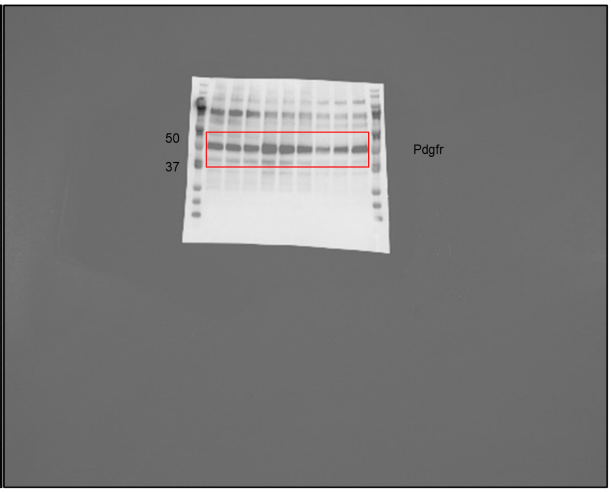

Et-1

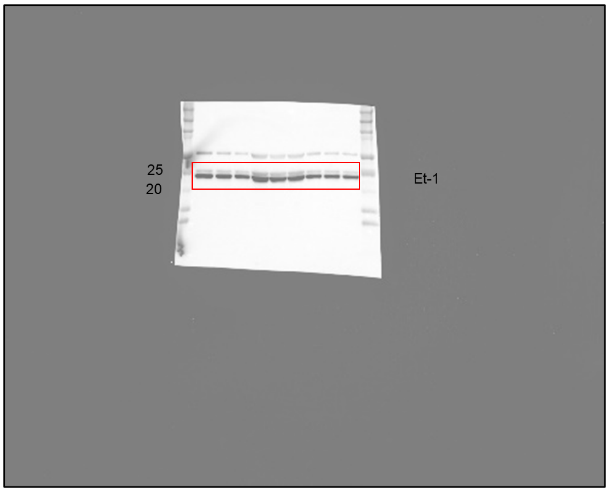

Figure 6

Gapdh

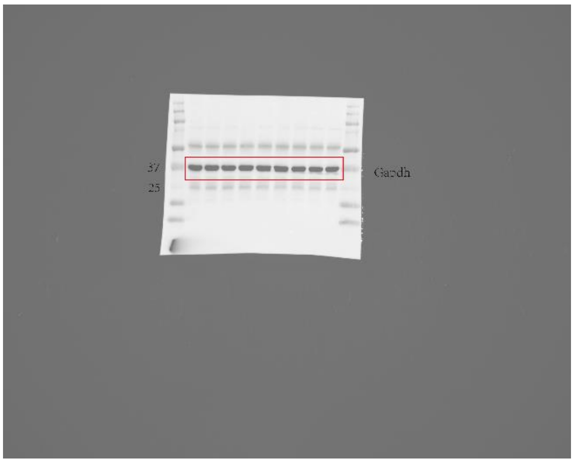

C3

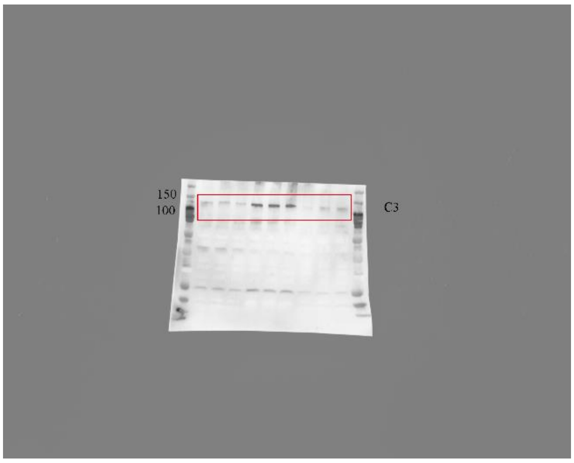

Pcna Tgf-β

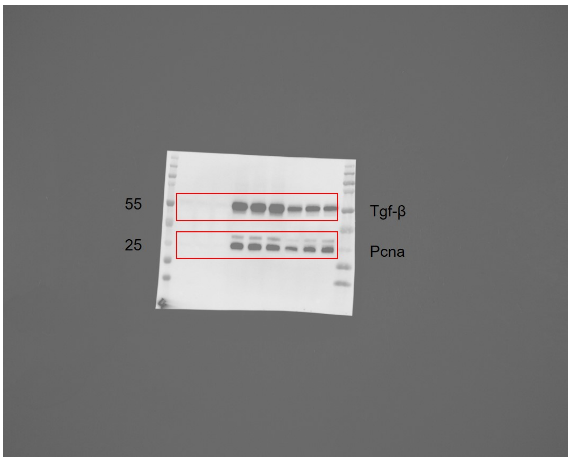

Et-1

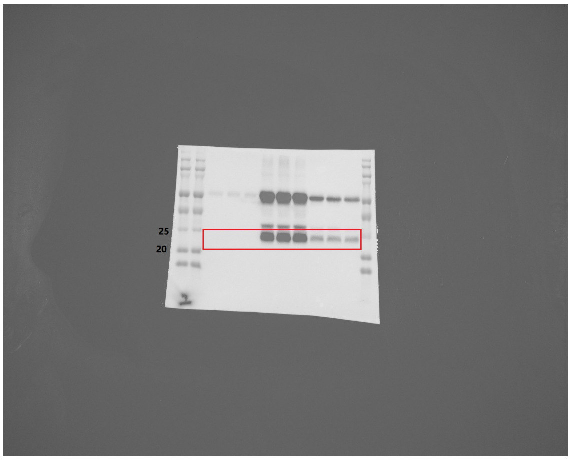

Cxcl12

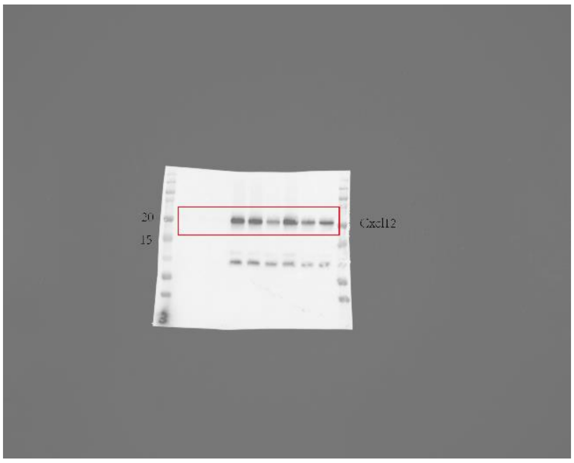

Pdgfrb

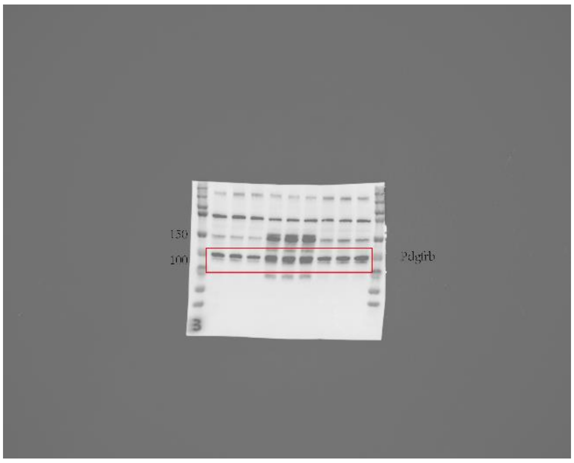

Figure S7

C3 Cxcr4

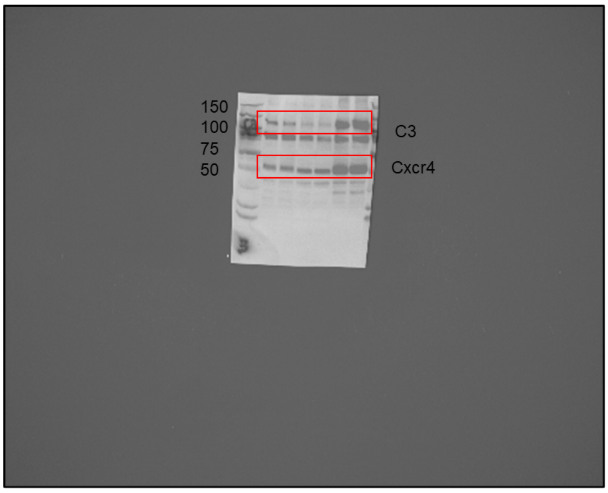

Figure S10

Gapdh

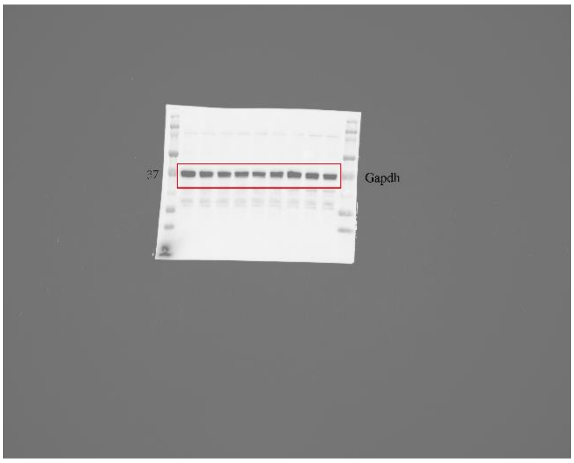

Cxcl12

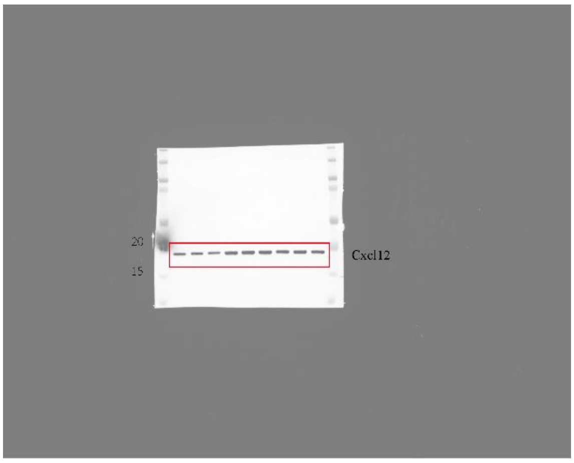

Tgf-β, Vimentin

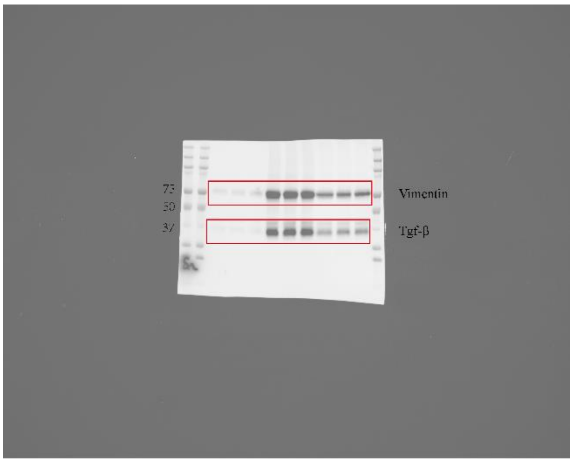

α-SMA

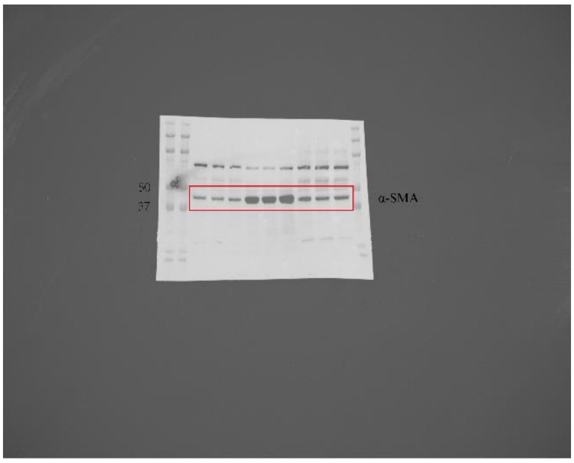

Collagen-1

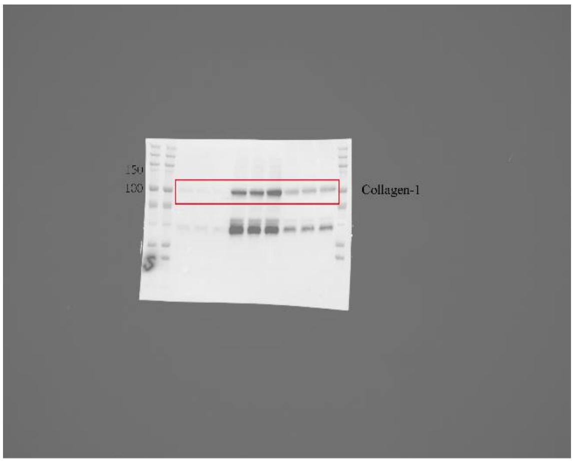

Fibronectin

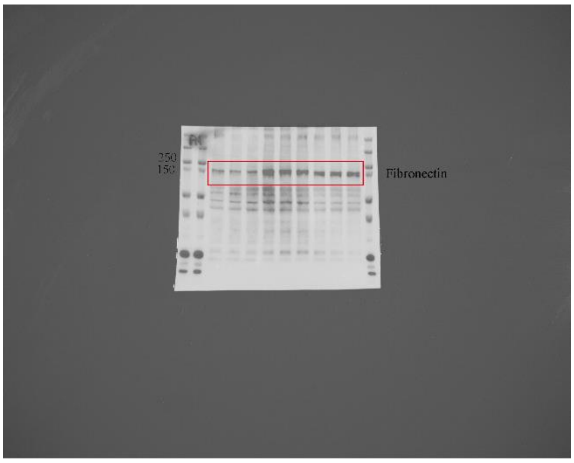

Supplement: Unedited blot and gel images [file jciinsight-9-173651-s028.pdf]
